# Activation-Induced Cytidine Deaminase Does Not Impact Murine Meiotic Recombination

**DOI:** 10.1534/g3.113.005553

**Published:** 2013-04-01

**Authors:** Catarina S. Cortesao, Raquel F. Freitas, Vasco M. Barreto

**Affiliations:** Instituto Gulbenkian de Ciência, 2780-156 Oeiras, Portugal

**Keywords:** AID, meiotic recombination, germline, cytidine deaminase, double-strand breaks

## Abstract

Activation-induced cytidine deaminase (AID) was first described as the triggering enzyme of the B-cell−specific reactions that edit the immunoglobulin genes, namely somatic hypermutation, gene conversion, and class switch recombination. Over the years, AID was also detected in cells other than lymphocytes, and it has been assigned additional roles in the innate defense against transforming retroviruses, in retrotransposition restriction and in DNA demethylation. Notably, AID expression was found in germline tissues, and in heterologous systems it can induce the double-strand breaks required for the initiation of meiotic recombination and proper gamete formation. However, because AID-deficient mice are fully fertile, the molecule is not essential for meiosis. Thus, the remaining question that we addressed here is whether AID influences the frequency of meiotic recombination in mice. We measured the recombination events in the meiosis of male and female mice F1 hybrids of C57BL/6J and BALB/c, in *Aicda^+/+^* and *Aicda^−/−^* background by using a panel of single-nucleotide polymorphisms that distinguishes C57BL/6J from BALB/c genome across the 19 autosomes. In agreement with the literature, we found that the frequency of recombination in the female germline was greater than in male germline, both in the *Aicda^+/+^* and *Aicda^−/−^* backgrounds. No statistical difference was found in the average recombination events between *Aicda^+/+^* and *Aidca^−/−^* animals, either in females or males. In addition, the recombination frequencies between single-nucleotide polymorphisms flanking the immunoglobulin heavy and immunoglobulin kappa loci was also not different. We conclude that AID has a minor impact, if any, on the overall frequency of meiotic recombination.

Genomes can be diversified through the recombination of genetic material. One example is the meiotic recombination that occurs during the process in which a diploid cell gives rise to haploid gametes (Gerton and Hawley 2005). In meiosis, two consecutive divisions segregate first the homologous chromosomes and then the sister chromatids. The events leading to a crossover start in prophase I, with the pairing of the homologous chromosomes in leptotene, which progresses through zygotene and is completed in pachytene. Subsequently, synapsis between the chromosomal axes of the paired homologous chromosomes is achieved by the formation of the synaptonemal complex. The critical enzyme for meiotic recombination is a topoisomerase II-like enzyme, Spo11, which introduces double-strand breaks (DSBs) throughout the genomic DNA in the leptotene to zygotene transition (Keeney *et al.* 1999; Romanienko and Camerini-Otero 1999; Inagaki *et al.* 2010) and in mammals is also involved in the formation of the synapsis (Baudat *et al.* 2000; Romanienko and Camerini-Otero 2000). Meiotic DSBs are not distributed at random in the genome but rather concentrated in hotspots (Smagulova *et al.* 2011).

In vertebrates, additional examples of genome diversification through recombination are found in the antigen receptor loci of lymphocytes, and most notably in the immunoglobulin genes of B-cells. During V(D)J recombination in developing lymphocytes, the RAG1/RAG2 complex assembles different sequence modules to produce a rearranged immunoglobulin, thus creating cells with unique specificities (Tonegawa 1983). When activated, these cells can then change the constant region of the antibody and its effector function through a reaction termed class switch recombination. The enzyme that triggers such recombination is activation-induced cytidine deaminase (AID or *Aicda* gene), a molecule that is also responsible for further editing of the rearranged immunoglobulin genes through somatic hypermutation and gene conversion (Muramatsu *et al.* 2000; Revy *et al.* 2000; Arakawa *et al.* 2002; Harris *et al.* 2002). The triggering event in all AID-dependent reactions is the deamination of cytidines in the exposed single strands of DNA of the transcription bubble, which become uracils and will eventually lead to the fixation of point mutations (somatic hypermutation), the introduction of a germline-encoded patch of nucleotides (gene conversion) or to the generation of DSBs (class switch recombination) (Petersen-Mahrt *et al.* 2002).

Although the physiological role of AID in the context of antibody formation and also its oncogenic potential have been thoroughly described, some reports suggest that the role of this enzyme is not confined to adaptive immunity (reviewed in Barreto and Magor 2011). AID transcripts were found in cells other than lymphocytes, most notably in mouse primordial germ cells and unfertilized oocytes (Morgan *et al.* 2004), and in testicular tissue from human biopsies (Schreck *et al.* 2006). It has been proposed that AID shares a role in retrotransposition restriction with other members of the AID/APOBEC family, but it has not been shown that AID plays such a role in the germline or early embryogenesis (MacDuff *et al.* 2009). It has also been observed that *Aicda^−/−^* mice have increased levels of methylated DNA in the primordial germ cells (Popp *et al.* 2010). This finding is consistent with the original idea of Morgan *et al.* (2004) that AID may function as a demethylase and with other reports of AID-dependent DNA demethylation (Rai *et al.* 2008; Bhutani *et al.* 2010; Guo *et al.* 2011), although 5-methylcytosine is a poor substrate for AID *in vitro* (Larijani *et al.* 2005; Wijesinghe and Bhagwat 2012). Finally, based on the observation that the expression of AID in *Schizosaccharomyces pombe* and *Caenorhabditis elegans* partially rescues the *rec12Δ* and Spo11-deficient phenotypes, respectively, it has been proposed that AID may contribute to meiotic recombination (Pauklin *et al.* 2009), but this hypothesis has not been tested *in vivo*.

Besides the rescue experiment suggesting that AID may introduce DSBs that lead to crossover events between homologous chromosomes, additional arguments strengthen the hypothesis that this cytidine deaminase could have a role in meiotic recombination: (1) AID was shown to mutate other regions of the genome besides the antigen receptor loci, making it a potential contributor to the overall frequency of meiotic recombination (Chiarle *et al.* 2011; Klein *et al.* 2011); (2) AID is more easily detected in oocytes than in the male germline (Morgan *et al.* 2004) and the AID-dependent demethylation in the primordial germ cells is stronger in females (Popp *et al.* 2010), which correlates with the known observation that meiotic recombination levels are greater in female mice than in males (Shifman *et al.* 2006); (3) the reactions involving AID and Spo11 share a number of features, from the H3k4me3 mark in the sequences where the DSB will be introduced to the subsequent recruitment of DNA repair pathways (Begum and Honjo 2012). However, given that meiotic recombination is an unavoidable step in gamete formation and that, unlike *Spo11^−^*^/−^ mice (Romanienko and Camerini-Otero 2000), *Aicda^−/−^* mice are fully fertile, AID does not have a fundamental role in meiosis. Thus, the question that we explore here is whether AID functions as a modifier that influences the frequency of meiotic recombination in mice.

## Materials and Methods

### Mice

*Aicda^+/−^* mice in C57BL/6J genetic background (B6.Aicda^+/−^, courtesy of Dr. Cristina Rada), *Aicda^+/−^* in BALB/c genetic background (BA.Aicda^+/−^, courtesy of Dr. Almudena Ramiro), F1 offspring, and C57BL/6J (received from The Jackson Laboratory and bred in the Instituto Gulbenkian de Ciência mouse facility) were housed and handled in accordance with the Instituto Gulbenkian de Ciência ethical committee.

### Meiotic recombination frequency detection and analysis

#### Sample selection:

B6.Aicda^+/−^ were crossed with BA.Aicda^+/−^ to generate F1.Aicda^−/−^ and F1.Aicda^+/+^ littermates, confirmed by standard genotyping polymerase chain reaction (PCR). From these animals, 18 stable reciprocal matings were set by crossing F1.Aicda^−/−^ or F1.Aicda^+/+^ with C57BL/6J (Supporting Information, Figure S1A). From the offspring of these reciprocal matings, tail biopsies were frozen for DNA extraction. Of these, 700 samples of DNA were extracted by isopropanol (Sigma-Aldrich) precipitation, and the origin and quality of DNA was confirmed by regular genotyping PCR. DNA from original mating pairs B6.Aicda^+/−^ and BA.Aicda^+/−^ were used as controls for single-nucleotide polymorphism (SNP) genotyping [B6.Aicda^+/−^ has 98% of C57BL/6J genetic background (n = 8); and BA.Aicda^+/−^ has 97.4% of BALB/c genetic background (n = 6)]. After SNP genotyping, only samples with more than 90% of detectable genotype signal (call) were considered for analysis.

#### SNP selection and genotyping:

A panel of 148 SNPs that distinguish between C57BL/6J and BALB/c genetic backgrounds was selected across the 19 autosomes to be evenly spaced with a physical distance of approximately 17 Mbp (based on the NCBI build36) between 2 consecutive SNPs on the same chromosome, using online tools (NCBI, MGI and Applied Biosystems), regardless of function (Table S1, Figure S1B). Multiplex SNP assays were designed using the Sequenom Platform Assay Design (SNP primers were ordered from Metabion, Germany), and SNP genotyping was performed on the Sequenom massARRAY iPLEX platform (Sequenom, San Diego, CA) using multiplexed amplification followed by mass-spectrometric product separation.

From the initial panel, only SNPs with more than 95% of detectable genotype signal (call) and SNPs with no distortion from the expected 50% inheritance of each allele on a 99% confidence interval were considered for analysis (Table S1). A total of 17 SNP/sample pairs passed the aforementioned filters but had no detectable genotype signal. To control for the effect of this absent data, we considered two extreme scenarios: recombination in all locations *vs.* no recombination in all locations; and overall recombination frequencies were calculated for the two extreme scenarios (Figure S2). Given that the statistical differences were similar in both, the values considered for further analysis were the ones substituted in a conservative manner (no recombination in that location). After applying these filters, 130 SNPs were used to distinguish between C57BL/6J from BALB/c genome, across the 19 autosomes with an average distance of 18.57 Mbp between 2 consecutive SNPs on the same chromosome (range, 6.93–74.98 Mbp) and a minimum resolution power of 34.62 Mbp (Table 1).

**Table 1:** Panel of 130 single-nucleotide polymorphisms (SNPs) across the 19 autosomes

Chr. Size	Chr. Size, Mbp^a^	No. SNPs	Distance between SNPs, Mbp^b^	Minimal Distance, Mbp^c^	Maximal Distance, Mbp^d^	Physical Length, Mbp^e^
1	197	13	15.62	8.69	30.36	187.5
2	182	7	28.93	9.93	74.98	173.6
3	160	8	20.78	11.37	46.28	145.5
4	156	9	18.08	11.81	31.86	144.7
5	153	9	17.89	8.17	44.27	143.1
6	150	6	27.31	12.45	61.62	136.5
7	153	9	16.59	12.31	33.07	132.7
8	132	9	14.71	9.69	20.33	117.7
9	124	6	16.56	10.73	30.78	82.8
10	130	5	26.05	13.03	48.46	104.2
11	122	7	18.62	6.93	30.59	111.7
12	121	4	23.64	12.64	31.87	70.9
13	120	7	17.32	13.58	28.97	103.9
14	125	8	13.91	8.98	20.84	97.4
15	103	5	15.12	12.74	16.28	60.5
16	98	5	17.54	10.62	30.33	70.2
17	95	5	21.05	7.05	30.49	84.2
18	91	4	14.17	11.89	16.51	42.5
19	61	4	17.15	7.97	29.87	51.4
Total	2473	130				2061.0
Average			18.57	10.56	34.62	

a Reported chromosome size in National Center for Biotechnology Information (NCBI).

b Distance between two consecutive SNPs, averaged for all SNPs per chromosome.

c Minimal distance between two consecutive SNP per chromosome.

d Maximal distance between two consecutive SNP per chromosome.

e The distance in Mbp between the first and the last SNP.

#### Meiotic recombination frequencies:

Meiotic recombination events occurring during meiosis of F1.Aicda^−/−^ or F1.Aicda^+/+^ were measured in the DNA of their offspring by crossing either F1 with C57BL/6J. In this way, for the expected signal per SNP genotype, one of the alleles always referred to the C57BL/6J parent and the other allele to the F1, which could either be of C57BL/6J or BALB/c origin. Thus, recombination events were defined as a change of SNP genotype signal from homozygous C57BL/6J to heterozygous or vice-versa between two consecutive SNPs on the same chromosome. Meiotic recombination frequencies were calculated using the genotyping results from 130 SNPs in 314 samples divided in four analysis groups corresponding to the offspring of the following matings: females F1.Aicda^−/−^ with male C57BL/6J (group termed FKO; n = 79); females F1.Aicda^+/+^ with male C57BL/6J (group termed FWT; n = 79); females C57BL/6J with males F1.Aicda^−/−^ (group termed MKO; n = 78); and females C57BL/6J with males F1.Aicda^+/+^ (group termed MWT; n = 78).

For a more detailed approach, six randomly chosen SNP pairs, two SNP pairs (in chromosome 19 for FWT *vs.* FKO analysis) for which in the first analysis the statistical difference was relatively high but not significant, and one SNP pair (in chromosome 17 for MWT *vs.* MKO analysis) for which the statistical analysis indicated a significant difference were used for genotyping an increased sample size. Thus, an additional 97 samples of DNA from FKO, 97 from FWT, 91 from MKO, and 97 from MWT were used for SNP genotyping (Table S2).

#### Variable number tandem repeat (VNTR) sequencing:

To test meiotic recombination frequencies flanking the IgH locus in chromosome 12, recombination frequencies were calculated using genotyping results from SNPs approximately at position 108 Mbp and using PCR results from a VNTR sequence that distinguishes C57BL/6J from BALB/c genome approximately at position 116 Mbp, assayed for offspring of FWT (n = 68), FKO (n = 67), MWT (n = 68), and MKO (n = 67). For this, PCR standard procedures with primers D12Mit134 (DNA segment, Chr 12, Massachusetts Institute of Technology 134)_F: 5′-CTATCTACAACAAACTTCTCCTGGG-3′ and D12Mit134_R: 5′-ACTCAGTCCAAACATATACAAGATGC-3′ were used.

### Sorting of subpopulations of cells according to DNA content in spermatogenesis

#### Sample preparation and staining:

Mouse testes were surgically removed from 8- to 12-wk-old B6.Aicda^+/+^ (n = 3) and B6.Aicda^−/−^ (n = 3). For testicular cell suspension, as previously described (Bastos *et al.* 2005), testes were meshed and seminiferous tubules were dissociated by collagenase I digestion (100 U/mL; Invitrogen) for 30 min at 32° in Solution-1 [HBSS (Invitrogen) supplemented with 20 mM HEPES (pH 7.2), 1.2 mM MgSO_4_.7 H_2_O, 1.3 mM CaCl_2_.2 H_2_O, 6.6 mM sodium pyruvate, and 0.05% lactate]. The cell suspension was then filtered through a 70-µm nylon mesh, centrifuged for 10 min at 400*g*, 4°, and the pellet was carefully ressuspended in Solution-2 [Solution-1 with L-glutamine (Invitrogen) and 1% fetal bovine serum (PAA Laboratories, Pasching, Austria)] and prepared for sorting. From the same mice, epididymis and *vas deferens* were removed to a Petri dish with Solution-1. Using forceps, the epididymis and *vas deferens* were pressed and meshed to release mature sperm cells. Cells were then washed, resuspended in Solution-2, counted, and processed for RNA extraction. To discriminate and sort subpopulations of testicular sperm cells according to DNA content, Hoechst 33342 (Ho; Invitrogen)—a vital dye that binds to DNA—was used as previously described (Bastos *et al.* 2005). For the staining, 20 million cells were incubated with 5 µg/mL Ho in 10 mL of Solution-2 for 90 min at 32°. Cells were then put on ice for at least 5 min, centrifuged for 15 min at 400*g*, 4°, and the pellet was carefully ressuspended in Solution-2 with propidium iodide (PI; 1 µg/mL; Invitrogen) to exclude dead cells.

#### Analysis and sorting:

Analysis and sorting was performed on a MoFlo (Beckman Coulter) equipped with a Coherent Innova I90C argon laser tuned to multiline UV (330−360 nm, 60-mW output) to excite Ho and a 488-nm Coherent Sapphire 488-200 CDRH laser (140-mW output) for forward and side scatter as well as for PI excitation. A 505-nm long-pass dichroic mirror (505DCXR; Chroma) was used to separate blue from red Ho fluorescence and to direct emitted light to different detectors. Ho blue was collected using a 440/20-nm band pass filter (Z440/20X; Chroma) and Ho red fluorescence with a 645-nm long pass filter (HQ645LP; Chroma). Fluorescence from PI was detected using a 670/40-nm band pass filter (D670/40; Chroma). Sorting was performed with a 70-µm nozzle at 414 kPa (60 psi) and with a ∼96 kHz Drop Drive frequency. Purity of sorted populations was assessed by analysis of sorted populations by flow cytometry immediately after the sort and also by analysis of DNA content in fixed sorted cells by PI incorporation. In summary, samples of different sperm cell populations were fixed with 80% ethanol and incubated with PI (30 µg/mL) and RNAase (30 µg/mL; Sigma-Aldrich) in phosphate buffered saline (Invitrogen) to check DNA content (Figure S4, A and B). Flow cytometric analysis was performed on a FACScan (Becton Dickinson). Analysis was performed using FlowJo software (Tree Star Inc). After sorting, testicular sperm cells 4N, 2N, N and also unsorted testicular sperm cells were immediately processed for RNA extraction.

#### Isolation of oocytes:

B6.Aicda^+/+^ and B6.Aicda^−/−^ females were given intraperitoneal pregnant mare serum gonadotropin (5 IU; Sigma-Aldrich) and 48 hours later were given intraperitoneal human chorionic gonadotropin (5 IU; Sigma-Aldrich) for super-ovulation. The next day oviducts were surgically removed, separated, and placed in a drop of M2 (Sigma-Aldrich). The *ampullae* (part of the oviduct) were tore using needles to release the super-ovulated oocyte structures that include the germ-cell surrounded by layers of granulosa cells. For “complete oocyte” samples, the oocytes with granulosa were briefly washed in M2 and mouth-pipetted to a different Petri dish with M2 and set aside for processing. For oocyte “granulosa free” samples, the oocytes with granulosa were incubated in M2 with hyaluronidase (150 µg/mL; Sigma-Aldrich) for 5 min at room temperature. Single oocytes were washed 3 times and set aside for processing and the remaining cells, considered “oocyte-free” granulosa cells, were carefully pipetted to a different Petri dish with M2, washed 3 times, and set aside for processing. As a control, ovaries from the super-ovulated females (“empty” ovaries) and from nonsuper-ovulated females were surgically removed, washed in M2, and meshed. All samples were immediately processed for RNA or protein extraction.

#### Splenocytes isolation and culture:

As controls for expression of *Aicda*, B-cells from spleens of B6.Aicda^+/+^ and B6.Aicda^−/−^ were stimulated in culture to up-regulate *Aicda* expression. In summary, homogenized single-cell splenocyte suspensions from 8- to 12-wk-old mice were depleted of red blood cells by lysis with ACK buffer (0.15 M NH_4_Cl, 10.0 mM KHCO_3_, and 0.1 mM ethylenediamine tetraacetic acid), washed twice, counted, and 1.5 × 10^6^ cells/mL were plated in 24-well plates in complete RPMI 1640 with Glutamax (Invitrogen) with 10% fetal bovine serum, penicillin and streptomycin (100 mM; Invitrogen), and 2-mercaptoethanol (50 µM; Invitrogen) supplemented with 20 mg/mL lipopolysacharide (Sigma-Aldrich) and interleukin-4 (supernatant from hybridoma clone X63, in-house production). After 3 d in culture, cells were washed, incubated for 5 min in TriPure Isolation Reagent (Tryzol; Roche), and stored at −80°.

#### Preparation of DNA for SNP genotyping and for regular PCR:

DNA was extracted from tail biopsies by proteinase K (Novagen, Germany) digestion and isopropanol (Sigma-Aldrich) precipitation. Mice were genotyped for *Aicda* locus using standard PCR procedures with primers FR312 5′-CCTAGTGGCCAAGGTGCAGT-3′ and FR313 5′-TCAGGCTGAGGTTAGGGTTCC-3′ for the wild-type allele and primers FR310 5′-GGCCAGCTCATTCCTCCACT-3′ and FR311 5′-CACTGAGCGCACCTGTAGCC-3′ for the knockout allele.

#### RNA extraction, complementary DNA (cDNA) synthesis, and real-time PCR:

All sorted and unsorted cell populations were further processed for RNA extraction using a commercial kit specific for small cell samples (Quick-RNA MicroPrep; Zymo Research) or for cells stored in Trizol (Direct-zol RNA MiniPrep; Zymo Research). Single-stranded cDNA was produced using random primers and SuperScript II reverse transcriptase (Invitrogen). cDNA from stimulated B-cells of B6.Aicda^+/+^and B6.Aicda^−/−^ was used as a control for the real-time PCR. *Aicda* mRNA was quantified using a SYBR green assay (Applied Biosystems). Beta-actin amplifications were used as normalization controls. The primers used were as follows: F_5-CCTAAGACTTTGAGGGAGTCAA-3 and R_5-CACGTAGCAGAGGTAGGTCTC-3 for *Aicda* and F_5-AGCTGTGCTATGTTGCTCTAGACTT-3 and R_5-CACACTTCATGATGGAATTGAATGTAG-3 for beta-actin. Quantitative PCRs were performed on 7900HT fast real-time PCR system (Applied Biosystems) using the following cycling program: 2 min at 50°, 10 min at 90° and 45 cycles of 95° for 15 sec and 60° for 1 min. Expression data of real-time PCR was averaged between triplicate replicas, calculated using Pfaffl ratio (Pfaffl 2001) and beta-actin as internal control.

#### Western blot:

Protein extraction was performed using NP40 Cell Lysis Buffer (Invitrogen) supplemented with protease inhibitor cocktail (Complete, Roche). Samples were run on a 4–12% sodium dodecyl sulfate polyacrylamide gel electrophoresis gel using the NuPAGE system (Life technologies) and semi-dry transferred to Imobilon-P membrane (Millipore). Membranes were blocked in 5% bovine serum albumin (Sigma-Aldrich) in Tris-Buffered Saline, 0.05% Tween-20 (TBS-T), stained with rabbit anti-AID serum (1:500 dilution, courtesy of Dr. Kevin McBride), and α-tubulin was used as loading control (anti-α-tubulin, 1:5000 dilution, Sigma-Aldrich). Signal was developed using horseradish peroxidase−conjugated antibodies (1:5000 dilution; Santa Cruz Biotechnology) and Super-Signal West Pico Chemiluminescent Substrate Kit per manufacturer instructions (Thermo Scientific).

#### Statistical analysis:

For the analysis of frequency of meiotic recombination between different groups, a Mann-Whitney test was used and statistical significance threshold is referred for each case. For the analysis of the proportion of recombination events for each SNP pair, the confidence intervals were calculated using the Agresti-Coull method and differences between wild type and knockout data were tested using a Fisher exact test. Standard Bonferroni correction on the *p* values was applied for multiple testing. All statistic tests were performed using GraphPad Prism (v5.00 Windows, GraphPad Software, San Diego California USA).

## Results

### AID does not affect global levels of recombination

**Figure 1:**
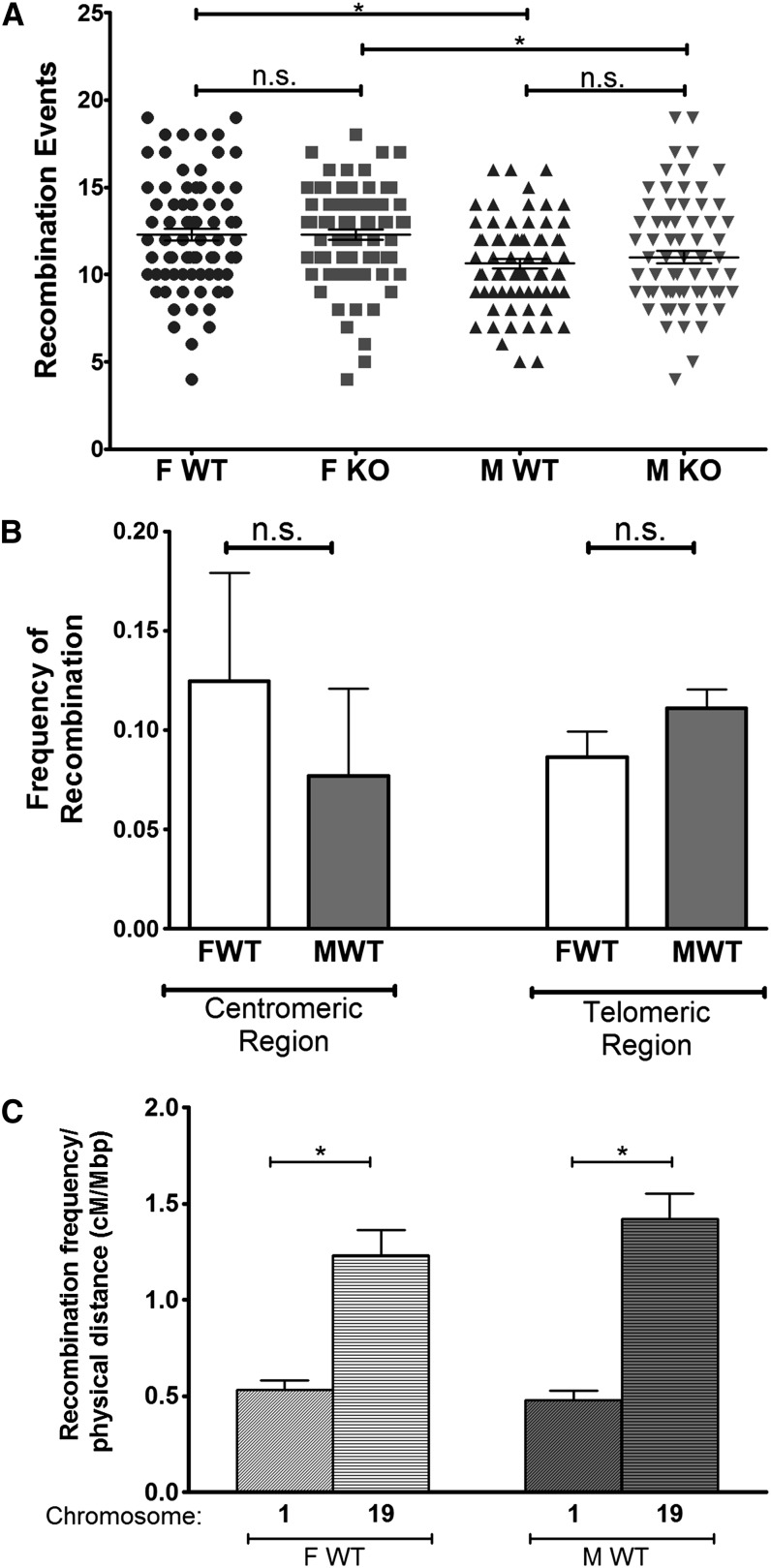
(A) Recombination events for all studied groups. The numbers of recombination events were calculated for all 19 autosomes per offspring of females F1.Aicda^+/+^ (FWT, n = 79), females F1. Aicda^−/−^ (FKO, n = 79), males F1. Aicda^+/+^ (MWT, n = 78), and males F1. Aicda^−/−^ (MKO, n = 78). Each dot represents a sample; lines represent mean ± SEM. A Mann-Whitney test was used to compare sample groups using a Bonferroni correction for multiple testing, * *P* < 0.0125; n.s., nonsignificant. (B) Average recombination events for centromeric and telomeric regions. For these analyses, only the SNP pairs closer to the regions were included. Thus, for the centromeric region only, the recombination frequencies of the first SNP pair in chromosomes 1, 7, 8, 11, 18, and 19 (n = 6) were used in the comparison between progeny of the female (FWT) and the male (MWT) F1.Aicda^+/+^. For the telomeric regions only, the recombination frequencies of the last SNP pair in chromosomes 1, 2, 3, 4, 5, 8, 9, 11, 14, 16, 17, and 19 (n = 12) were used in the comparison between the progeny of the female (FWT) and the male (MWT) F1.Aicda^+/+^. Bar and bar error represent mean ± SEM. A Mann-Whitney test was used to compare sample groups using a Bonferroni correction for multiple testing, * *P* < 0.0125; n.s., nonsignificant. (C) Average recombination events per physical size (Mbp/cM) for Chromosomes 1 and 19 for F1.Aicda^+/+^ progeny. Bar and bar error represent mean ± SEM. A Mann-Whitney test was used to compare FWT *vs.* MWT groups in each chromosome, *P < 0.05.

The documented expression of AID in gametes, reproductive organs, and pluripotent tissues, (Morgan *et al.* 2004; Schreck *et al.* 2006; MacDuff *et al.* 2009; Bhutani *et al.* 2010); its activity in primordial germ cells (Popp *et al.* 2010); and the observation that the heterologous expression of AID can partially rescue meiotic recombination in *S. pombe* and *C. elegans* (Pauklin *et al.* 2009) raised the question of whether normal levels of endogenous AID can influence the frequency of meiotic recombination in mice. To test this, recombination frequencies in the germline of male or female *Aicda^+/+^* or *Aicda^−/−^* (C57BL/6JxBALB/c)F1 genetic background were inferred by SNP genotyping of genomic DNA from the offspring of reciprocal matings of F1.Aicda^+/+^ or F1.Aicda^−/−^ with C57BL/6J (Figure S1A). For the expected signal per SNP genotype, one of the alleles referred to the C57BL/6J parent and the other allele to the F1, which could either be of C57BL/6J or BALB/c origin. Recombination events were then defined as a change of genotype signal (from homozygous to heterozygous or vice-versa) between two consecutive SNPs on the same chromosome. We found a total of 3629 recombinations across the 19 autosomes of the 314 animals included in the four analysis groups. Because the detection of virtually all crossovers would require a 10-Mbp resolution (Paigen *et al.* 2008) and our analysis has a 34-Mbp resolution, we first compared the number of recombination events calculated for F1.Aicda^+/+^ with the one described in larger studies. The estimated total number of sex-averaged recombination events per meiosis according to data from Shifman *et al.* (2006) and Paigen *et al.* (2008) is 13.9, and we observe 11.5 sex-averaged recombination events per meiosis. Thus, a small collection of SNPs is sufficient to detect around 83% of all recombination events, most likely due to the positive crossover interference in the murine meiosis (Broman *et al.* 2002). The comparison of the frequencies of recombination per chromosome we observed with those from a larger study (Shifman *et al.* 2006) shows that we are detecting the vast majority of recombination events in some chromosomes (Table S3), which is confirmed by a similar distribution of the number of recombination events in chromosome 1 (*P* = 0.88 for females; *P* = 0.13 for males by χ^2^) when we compare our data with data from Paigen *et al.* (2008). We then asked whether detecting the majority of crossover events is sufficient to capture a number of general features of meiotic recombination, such as the differences due to the gender of the transmitting parent, chromosomal location, and the physical size of the chromosome (Jensen-Seaman *et al.* 2004). The average number of recombination events in all of the 19 autosomes, averaged per mouse, showed that the frequency of recombination in the female germline was greater than in male germline (12.29 ± 0.35 for female and 10.63 ± 0.27 for male, *P* = 0.0007, Figure 1A, Table 2), which is consistent with the literature (Jensen-Seaman *et al.* 2004; Lynn *et al.* 2005; Shifman *et al.* 2006), and it is also observed for the F1.Aicda^−/−^ study groups (12.29 ± 0.30 for female and 11.00 ± 0.34 for male, *P* = 0.0008). We then compared recombination frequencies according to position in the chromosome, and, although the differences are not statistically significant, we found the expected trends toward greater recombination frequencies in the male germline near the telomeres and the inverse for the recombination frequencies near the centromeres (Figure 1B) (Shifman *et al.* 2006). Finally, when we pooled the female and male recombination frequencies between chromosome 1 (size 197 Mbp) and 19 (size 61 Mbp), we found at least a twofold increase in the smallest chromosome compared with the biggest, an inverse correlation between chromosomal size and average recombination frequencies in the mouse genome that is well-documented (Figure 1C) (Shifman *et al.* 2006). Overall, these observations indicate that, although we miss 17% of the recombination events reported in larger studies, detecting the majority of the crossovers is sufficient to capture global features of meiotic recombination.

**Table 2:** Number of recombinations detected per chromosome

Chromosome Number	Sample Groups							
	FWT (n = 79)		FKO (n = 79)		MWT (n = 78)		MKO (n = 78)	
	No. Rec*^a^*	cM/Mbp*^b^*	No. Rec*^a^*	cM/Mbp*^b^*	No. Rec*^a^*	cM/Mbp*^b^*	No. Rec*^a^*	cM/Mbp*^b^*
1	79	0.53	68	0.46	70	0.48	65	0.44
2	83	0.61	79	0.58	76	0.56	68	0.50
3	63	0.55	63	0.55	48	0.42	54	0.48
4	74	0.65	65	0.57	50	0.44	49	0.43
5	63	0.56	66	0.58	59	0.53	66	0.59
6	59	0.55	54	0.50	51	0.48	55	0.52
7	66	0.63	62	0.59	47	0.45	55	0.53
8	50	0.54	56	0.60	42	0.46	48	0.52
9	38	0.58	39	0.60	34	0.53	36	0.56
10	50	0.61	46	0.56	36	0.44	41	0.50
11	59	0.67	59	0.67	57	0.65	61	0.70
12	28	0.50	40	0.71	25	0.45	27	0.49
13	61	0.74	45	0.55	32	0.39	34	0.42
14	35	0.45	41	0.53	37	0.49	41	0.54
15	25	0.52	19	0.40	25	0.53	16	0.34
16	24	0.43	42	0.76	34	0.62	31	0.57
17	47	0.71	56	0.84	39	0.59	57	0.87
18	17	0.51	22	0.66	10	0.30	4	0.12
19	50	1.23	49	1.21	57	1.42	50	1.25
Total	971		971		829		858	

a Sum of the recombinations per chromosome.

b Recombination frequency divided by the physical length (see Table 1).

We then compared the overall recombination frequency between *Aicda^−/−^* and *Aicda^+/+^* and found no statistical difference in the female (*P* = 0.6155) or male (*P* = 0.8192) groups (Figure 1A). The same analysis including only chromosomes for which we detect the vast majority of recombination events, leads to loss of statistical significance in the female *vs.* male comparison and no difference in the recombination frequency of *Aicda^−/−^* (data not shown). In conclusion, our results show that AID does not have a major impact on the global frequency of meiotic recombination.

### Recombination frequencies per chromosome and SNP pair

**Figure 2:**
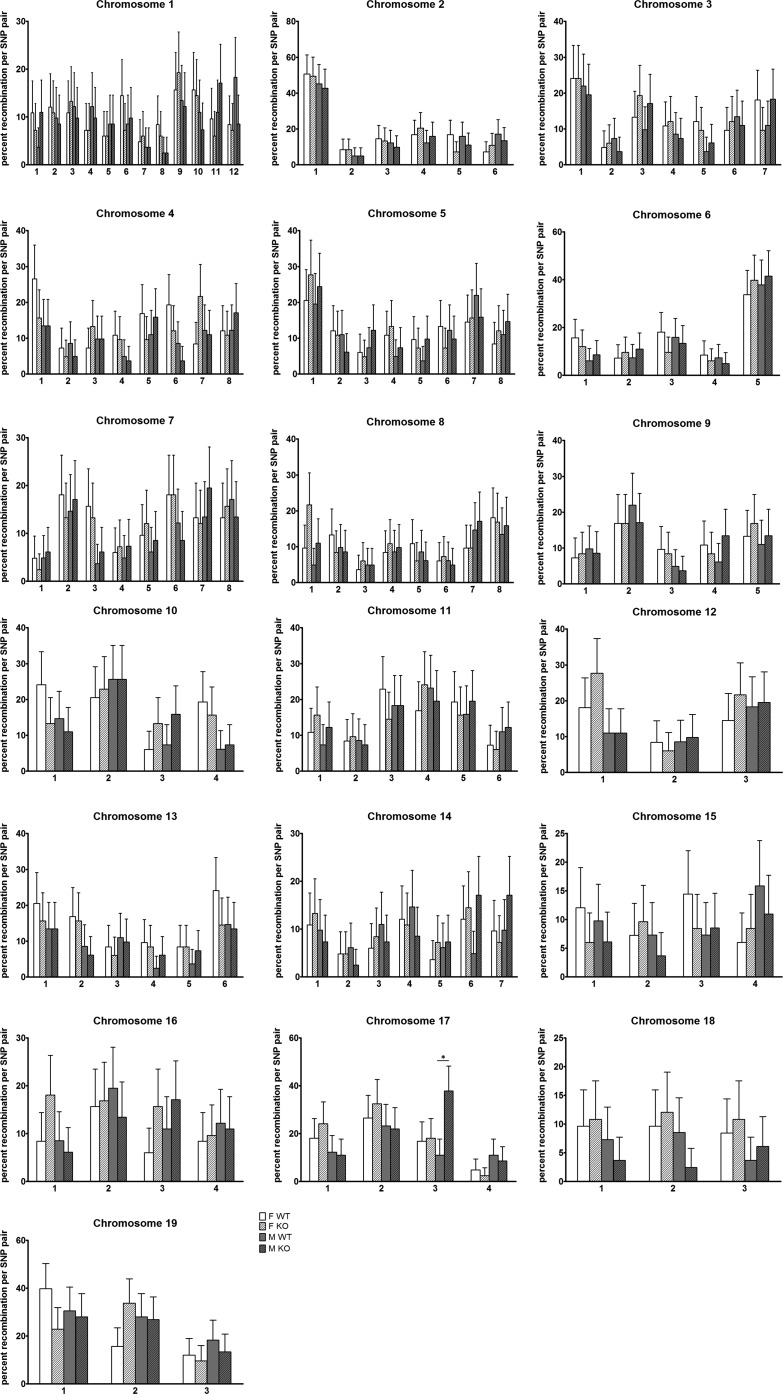
Proportions of recombination events per SNP pair for all 19 autosomes calculated for offspring of females F1.Aicda^+/+^ (FWT, n = 79, white bars), females F1.Aicda^−/−^ (FKO, n = 79, white bars with stripes), males F1.Aicda^+/+^ (MWT, n = 78, gray bars), and males F1.Aicda^−/−^ (MKO, n = 78, gray bars with stripes). On the x-axis, the order of the SNP pairs is shown. Error bars correspond to 95% confidence intervals as estimated by the Agresti-Coull method. A Fisher-exact test was used to compare sample groups using a Bonferroni correction for multiple testing, *P < 0.000225.

The recombination frequencies were analyzed per chromosome and plotted in an (x, y) scatter plot for comparison, between *Aicda^+/+^ vs. Aicda^−/−^* in female and male groups (Figure S3). A bisector line (represented as a dashed line) corresponds to equal recombination frequencies between the FWT and FKO or MWT and MKO analysis groups and deviations from this line, even if not significant, were observed for females in Chromosomes 12, 13, and 16 and for males in Chromosome 17. To further analyze this, recombination frequencies were compared per SNP pair (Figure 2) for the analysis groups of both female and male parents, *Aicda^+/+^ vs. Aicda^−/−^*, and no statistical significant differences were found, except for the comparison between MWT *vs.* MKO in Chromosome 17 (SNP pair in approximate position 68 Mbp and 86 Mbp), where there is a statistically significant increase in the recombination frequency in MKO compared with MWT. This SNP pair, two SNP pairs (in chromosome 19 for FWT *vs.* FKO analysis) for which in the first analysis the statistical difference was relatively high but not significant, and six randomly chosen SNP pairs were then used to genotype an additional set of samples. For the SNP pairs in chromosomes 17 and 19 (as for the other repeated SNP genotyping), results show that increasing the sample size does not lead to statistically significant differences and that the statistically significant difference from the previous set of samples is now lost (Table S2). Finally, because SNP pairs with low number of recombination events are less likely to include two recombination events in the same region than SNP pairs with high number of recombination events, which would not have been quantified in our system, we repeated the overall analysis using only SNP pairs with proportions of recombination events below a cut off of 10% or 5%, but this did not reveal any increase in the frequency of meiotic recombination in *Aicda^+/+^* compared to *Aicda^−/−^* (data not shown).

### Recombination frequencies of SNPs flanking immunoglobulin (Ig) genes

**Figure 3:**
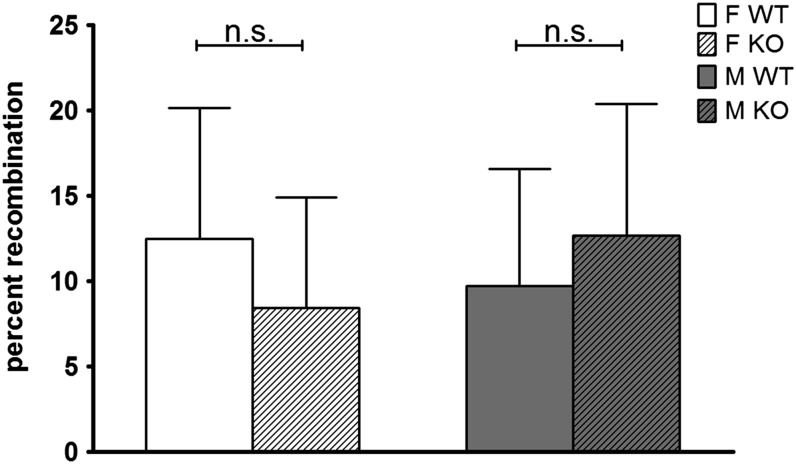
Average recombination frequencies of the IgH locus (chromosome 12), a putative AID target. Recombination frequencies were calculated using genotyping results from SNP in approximate position 108 Mbp and PCR results from VNTR sequence that distinguishes C57BL/6J from BALB/C genome in approximate position 116 Mbp calculated for offspring of FWT (n = 68), FKO (n = 67), MWT (n = 68), and MKO (n = 67). Bar and bar error represent mean ± SEM. A Mann-Whitney test was used to compare sample groups using a Bonferroni correction for multiple testing, *P < 0.0125; n.s. is non-significant.

We looked for the average recombination frequencies of the natural AID targets. AID-dependent mutations are found in the Ig loci at a frequency at least two orders of magnitude higher than in the remainder of the genome. In the IgH locus, which is the most complex of the antigen receptor loci, as it is a target for both somatic hypermutation and class switch recombination, recombination frequencies were calculated using genotyping results from SNPs approximately at position 108 Mbp and using PCR results from a VNTR sequence that distinguishes C57BL/6J from BALB/c genome approximately at position 116 Mbp and no differences in recombination frequencies were found (Figure 3). For the Igκ (in Chromosome 6), using flanking SNPs, no difference in recombination frequencies was detected. Other putative targets of AID mutagenic potential such as *c-myc* (chromosome 15) were also included in the analysis and no difference in recombination frequencies was found (Figure 2).

### *Aicda* expression in murine germline cells

Because we found no interference of AID activity in meiotic recombination, we decided to readdress whether *Aicda* transcripts can be detected in the germline. Morgan *et al.* (2004) reported no *Aicda* expression in mouse testes but high expression in ovaries and even higher in unfertilized oocytes; however, the authors of another study suggest that the expression of an *Aicda* reporter is observed only in somatic cells surrounding the oocyte (Qin *et al.* 2011) and Schreck *et al.* (2006) reported expression in testis tissue from human biopsies. These discrepancies may result from the methods used and/or reflect species differences between mouse and humans (Morgan *et al.* 2004; Schreck *et al.* 2006; Qin *et al.* 2011). We focused on the expression of *Aicda* in sorted populations of sperm cells, including the cells undergoing meiosis, and in isolated (postmeiotic) oocytes from adults and additional cells from the ovary.

**Figure 4:**
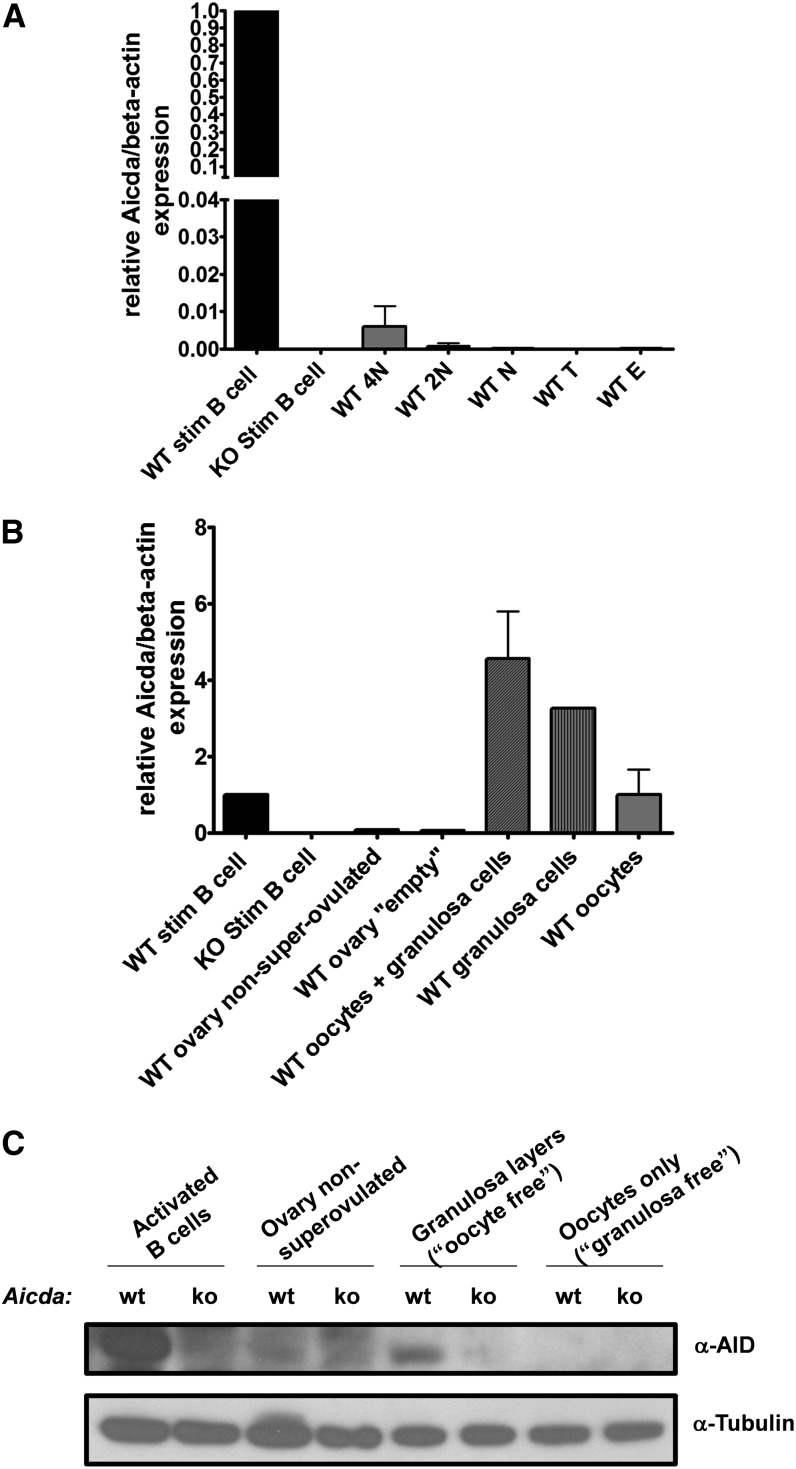
(A) *Aicda*/beta-actin expression levels measured by real-time PCR in activated B-cells of B6.Aicda^+/+^ and B6.Aicda^−/−^, B6.Aicda^+/+^ sorted 4N, 2N, and N (WT 4N, 2N, and N, respectively) spermatocytes, and unsorted testicular (WT T) and epididymal (WT E) sperm cells. (B) *Aicda*/beta-actin expression levels measured by real-time PCR in activated B-cells of B6.Aicda^+/+^ and B6.Aicda^−/−^, and B6.Aicda^+/+^ ovaries from non-superovulated females (WT ovary-non-superovulated), “complete oocyte” samples (WT oocytes + granulosa cells); only cells of the granulosa “oocyte free” (WT granulosa cells) and isolated oocytes (WT oocytes). Error bars correspond to two independent experiments in which the tissues were pooled from 16−20 females per sample. (C) Western blot analysis of activated B-cells of B6.Aicda^+/+^ and B6.Aicda^−/−^, B6.Aicda^+/+^ ovaries from non-superovulated females (WT ovary non-superovulated), “complete oocyte” samples (WT oocytes + granulosa cells); only cells of the granulosa “oocyte free” (WT granulosa cells) and isolated oocytes (WT oocytes).

As a consequence of ongoing spermatogenesis, the germinal population in the testis is highly heterogeneous with premeiotic, meiotic, and haploid postmeiotic cells and using the whole organ for expression studies may not reflect the true *Aicda* expression in homogeneous populations of cells at different stages of differentiation. Thus, testicular sperm cells were sorted based on DNA content (4N, 2N, and N) and *Aicda* transcripts were quantified by real-time PCR (Figure 4A; Figure S4, A and B). In the haploid (N) or diploid (2N) cell populations, these being the most prevalent in testis tissue, no *Aicda* expression was detected. In addition, no *Aicda* expression was detectable in nonsorted total testis tissue and in maturing sperm removed from the epididymis. In the 4N cell population, which is enriched in gametocytes that are in prophase I of meiosis, after chromosome duplication, *Aicda* expression was detectable, but at vestigial levels when compared to *Aicda* expression in activated B-cells (Figure 4A).

For female gametes, we did not look at the stage and tissues during embryonic development when meiosis takes place but simply investigated whether AID is expressed in the germline of adult mice. Oocytes were manually separated from the granulosa cells upon hyaluronidase digestion, to discriminate populations. *Aicda* expression was then tested in isolated oocytes, granulosa cells (“oocyte free”), oocytes with granulosa (follicles) and, as controls, whole ovaries post-superovulation (“empty”) and whole ovaries from animals that did not undergo superovulation. In isolated oocytes, *Aicda* expression was similar to activated B-cells and in the samples of “complete oocytes” and granulosa only (oocyte-free) *Aicda* expression was around four-fold that of the activated B-cells; the lower proportion of RNA coming from oocytes compared with that of granulosa in the combined sample probably explains the combined sample not having a lower expression of *Aicda* than the granulosa alone (Figure 4B). The transcript in whole ovaries was almost undetectable both in superovulated and non-superovulated samples. The effect of the hormones used to induce superovulation on the expression of AID was also tested under different activation conditions of splenic B-cells from superovulated and non-superovulated animals, and under the absence or presence of pregnant mare serum gonadotropin and human chorionic gonadotropin in the culture medium. Superovulation did not prime B-cells for greater *Aicda* expression upon activation and the effect of the hormones in culture was not always consistent, although we cannot exclude that certain conditions may enhance *Aicda* by two-fold (Figure S5). In any case, levels of *Aicda* transcripts are higher in the granulosa cells than in oocytes and, although in oocytes the transcript levels are similar to those of activated B-cells, the protein was only detected in the granulosa (Figure 4C).

## Discussion

Here we show that AID does not have a major impact in the frequency of meiotic recombination in mice, despite being detected in the germline (Morgan *et al.* 2004; Schreck *et al.* 2006) and previous observations that, when ectopically expressed in heterologous systems, this molecule produces DNA lesions that partially compensate for the absence of the natural DSB inducer in meiosis, Spo11 (in *C. elegans*) or Rec12 (*S. pombe*) (Pauklin *et al.* 2009). Our sample of genotyped animals and the collection of SNPs used are of modest size; however, the results for *Aicda^+/+^* capture a number of essential features of a larger dataset (Jensen-Seaman *et al.* 2004; Shifman *et al.* 2006): (1) we detect the sex-specific differences in the frequency of meiotic recombination; (2) we find an inverse relation between chromosome size and frequency of recombination; (3) we reproduce the finding that recombination rates are greater near the centromeres for females and toward the telomeres for males. Thus, although we cannot exclude that the inclusion of more animals and/or more SNPs in the analysis could reveal a role for AID in meiotic recombination, based on our data we estimate that its contribution, if any, is small. In addition, we cannot formally exclude that increasing the number of SNPs would not bring to view regions in which AID has an impact, but it is worth noticing that when we increased the size of the sample for the pairs of SNPs that had the most pronounced differences, the statistical significance decreased. Finally, our data clearly show that AID does not determine the sex-specific differences in the recombination frequency. Although a number of causes for this difference were already proposed (*e.g.*, Lercher and Hurst 2003; Lenormand and Dutheil 2005; Petkov *et al.* 2007), an additional role for AID was a possibility, given the differences in the AID expression levels detected in the male and female gametes, which are consistent with a slightly more pronounced increase in the methylation levels of the genome from primordial germ cells in *Aicda^−/−^* females than in the males (Popp *et al.* 2010).

At first glance, arguing for a role of AID in the germline is counterintuitive because this molecule is a potent mutator of immunoglobulin genes in the context of the immune response. However, error-prone repair of the AID-induced lesions seems to be characteristic of these target genes because the bulk of the off-target lesions are faithfully removed by the combined action of mismatch and base excision repair (Liu *et al.* 2008). How this is achieved is not fully understood, but it is reasonable to assume that, in the germline, high-fidelity repair would deal with most of AID-induced lesions. Interestingly, homologous recombination, the repair pathway involved in meiotic crossover, is also actively protecting the genome of lymphocytes against the activity of AID (Hasham *et al.* 2010). Furthermore, the AID-dependent demethylation observed in murine primordial germ cells argues for an activity of the molecule in the germline (Popp *et al.* 2010). Thus, knowing from the work of Pauklin *et al.* (2009) that the AID-induced lesions can be intermediates in the reaction leading to crossovers, why is AID not making a sizable contribution to meiotic recombination in mice? The expression level of the molecule during meiosis and the availability of the target sequences are the most trivial explanations.

Physiological levels of AID in the germline may not be sufficient to drive detectable meiotic recombination. It is not known whether AID is expressed in cells undergoing meiosis in female mice, but we have sorted different sperm cell populations and we were unable to detect AID expression. The only signal we found was in primary spermatocytes, which is consistent with what has been reported for the human testis (Schreck *et al.* 2006), but the levels we detected by real-time PCR are extremely low compared with the expression levels in activated B-cells.

In addition, it is possible that the cell-cycle regulation of AID activity and AID’s specific requirement for transcription limit the impact of this molecule in meiotic recombination. AID-induced DSBs and Nbs1/gamma-H2AX foci in the switch regions of the IgH locus are observed in the G1 phase of cells undergoing class switch recombination (Petersen *et al.* 2001; Schrader *et al.* 2007), similarly to point mutations in the V region (Faili *et al.* 2002). Thus, AID lesions are introduced and repaired in G1, although for the lesions that evade the G1/S checkpoint, repair occurs in S phase (Hasham *et al.* 2012). In any case, there are no reports of AID-induced lesions in G2 or during mitosis and, unless the cell-cycle regulation of AID in the germline is peculiar, probably AID is not active in prophase I, when recombination takes place. Furthermore, it is known that AID only acts on single stranded DNA, which can be exposed during transcription (Chaudhuri *et al.* 2003), but transcription is low in the early stages of the first meiotic prophase (Page *et al.* 2012). This does not imply that AID could not have access to target sequences exposed as single-stranded DNA in some transcription-independent manner, it simply suggests that the level of potential targets at that particular stage is probably lower than in a G1 cell.

Investigating whether AID-dependent crossovers can occur with the low frequency of the off-target AID-dependent DSBs found in activated B-cells by high throughput methods (Chiarle *et al.* 2011; Klein *et al.* 2011) does not seem fruitful. Thus, we conclude that AID has no sizable contribution for meiotic recombination. This finding shows that Spo11 and AID have nonoverlapping functions because *Spo11*^−/−^ mice also show normal immunoglobulin somatic hypermutation and class switch (Klein *et al.* 2002).

In agreement with a study using a lineage tracer reporter (Qin *et al.* 2011), we detected for the first time that the AID protein is expressed in the granulosa cells but not in the oocyte, despite being highly transcribed in both cell types. These findings suggest the possibility of two nonessential and largely unaddressed roles played by AID. First, AID transcripts may be stored in the oocyte for translation after fertilization, which could associate this gene to a maternal-effect. Second, given the intercellular communication between these cell types, the allocation of the AID protein expression to the surrounding granulosa cells may allow the enclosed oocyte to be protected against exogenous DNA and endogenous retroelements without being exposed to the direct mutagenic effects of AID.

## Supplementary Material

Supporting Information
