# Supplementary material for: Activation-Induced Cytidine Deaminase Does Not Impact Murine Meiotic Recombination
Source: G3 (Bethesda). 2013 Apr 1;3(4):645–55. doi: 10.1534/g3.113.005553 (PMC3618351; doi:10.1534/g3.113.005553)
Supplement: Supporting Information [file supp_3_4_645__index.html]

Activation-Induced Cytidine Deaminase Does Not Impact Murine Meiotic Recombination — Supporting Information 

# Activation-Induced Cytidine Deaminase Does Not Impact Murine Meiotic Recombination

## Supporting Information for Cortesao, Freitas, and Barreto, 2013

**Files in this Data Supplement:**

- Supporting Information - Figures S1-S5 and Tables S1-S3 (PDF, 1.4 MB)
- Figure S1 - (A) Schematics of the mating strategy (B) Schematics of the SNP relative positions on the 19 autossomes in black (PDF, 294 KB)
- Figure S2 - For the 17 SNP/sample pairs that passed the selection filters but had no detectable genotype signal, two extreme scenarios (recombination in all locations versus no recombination in all locations) were considered and overall recombination frequencies were calculated for the two extreme scenarios (PDF, 287 KB)
- Figure S3 - Representation of the average recombination frequency per chromosome of FWT vs FKO and MWT vs MKO study groups (PDF, 174 KB)
- Figure S4 - (A) Sorting strategy for subpopulations of testicular sperm cells according to DNA content using Hoechst 33342 - a vital dye that binds to DNA � as previously described (BASTOS et al. 2005). (B) Purity of the sorted populations, measured by acquisition of the subpopulations after sorting (left) and by DNA content measured by PI incorporation (right) and in the table the percentages of purity achieved for all the samples used in the real-time PCR. (PDF, 497 KB)
- Figure S5 - To control for the effect of the hormone stimulation on AID expression, splenic B-cells from super-ovulated and from non-super-ovulated C57BL/6J were stimulated in culture with LPS and under different IL4 concentrations (++ is double concentration of +), with or without PMSG (2.5 and 0.5 IU/ml) and HCG (2.5 and 0.5 IU/ml) or both in the culture medium (PDF, 156 KB)
- Table S1 - Panel of the initial SNPs and of the 130 used SNPs and the calculated distances between the SNP pairs used for the calculation of the recombination frequencies (PDF, 83 KB)
- Table S2 - Independent data sets to evaluate first results for recombination between SNP pairs (PDF, 50 KB)
- Table S3 - Comparison of the percentage of detected recombination events per chromosome in this study compared to an exhaustive analysis (PDF, 51 KB)
