# Supplementary material for: Activation-Induced Cytidine Deaminase Does Not Impact Murine Meiotic Recombination
Source: G3 (Bethesda). 2013 Apr 1;3(4):645–55. doi: 10.1534/g3.113.005553 (PMC3618351; doi:10.1534/g3.113.005553)
Supplement: Supporting Information [file supp_g3.113.005553_00553SI.pdf]

G3: Genes | Genomes | Genetics

Activation-Induced Cytidine Deaminase does not impact on murine meiotic recombination

Catarina S. Cortesao *, Raquel F. Freitas *, Vasco M. Barreto *

* Instituto Gulbenkian de Ciência, 2780-156 Oeiras, Portugal

DOI: 10.1534/g3.113.005553

Figure S1. A

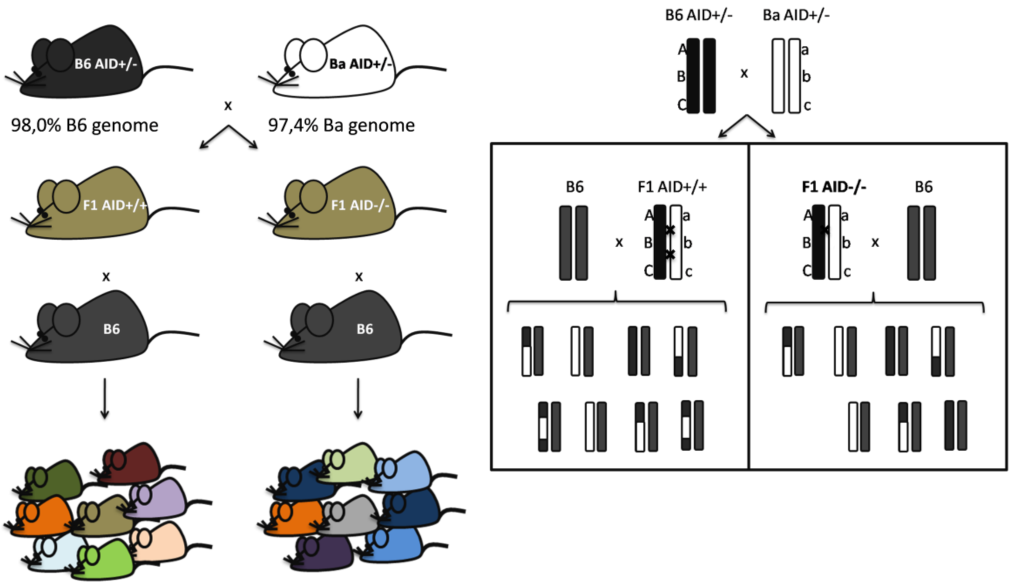

Figure S1. B

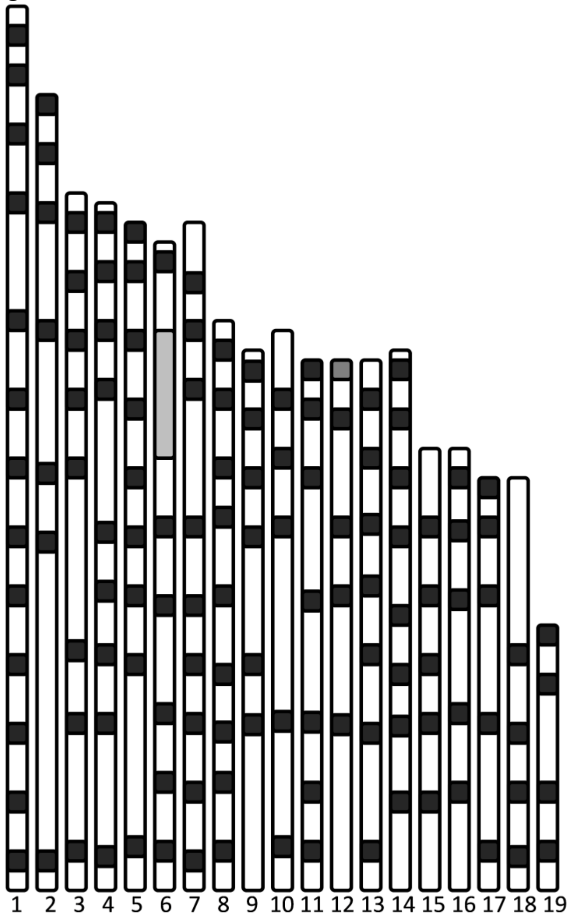

Figure S1 (A) Schematics of the mating strategy : matings of B6.Aicda+/- and BA.Aicda+/-; F1 were genotyped for Aicda locus. F1.Aicda-/- and F1.Aicda+/+ were reciprocally mated with C57BL/6J and offspring was genotyped for SNPs that distinguish C57BL/6J from BALB/c genetic background. (B) Schematics of the SNP relative positions on the 19 autossomes in black. In Chromosome 6, the light grey area represents the portion that contains the Aicda locus and that is still of 129 genome. In medium grey is represented the relative position of the Variable Number Tandem Repeat (VNTR) in chromosome 12.

Figure S2.

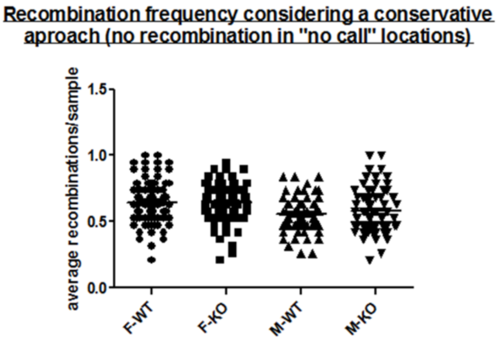

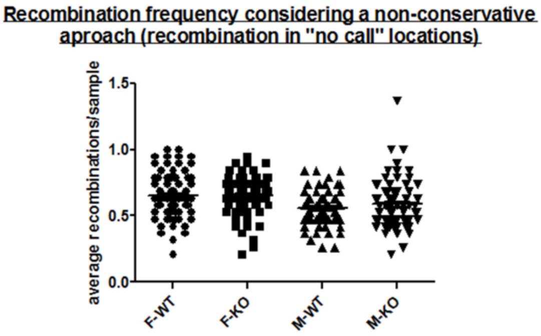

ManWhitey Test	Fwt no rec	Fko no rec	Mwt no rec	Mko no rec
Fwt no rec	na	0,6155	0,0007	0,0046
Fko no rec	na	na	< 0,0001	0,0008
Mwt no rec	na	na	na	0,8192
Mko no rec	na	na	na	na

ManWhitey Test	Fwt rec	Fko rec	Mwt rec	Mko rec
Fwt rec	na	0,6632	0,0004	0,0046
Fko rec	na	na	< 0,0001	0,0008
Mwt rec	na	na	na	0,7264
Mko rec	na	na	na	na

Figure S2 For the 17 SNP/sample pairs that passed the selection filters but had no detectable genotype signal, two extreme scenarios (recombination in all locations versus no recombination in all locations) were considered and overall recombination frequencies were calculated for the two extreme scenarios.

Figure S3.

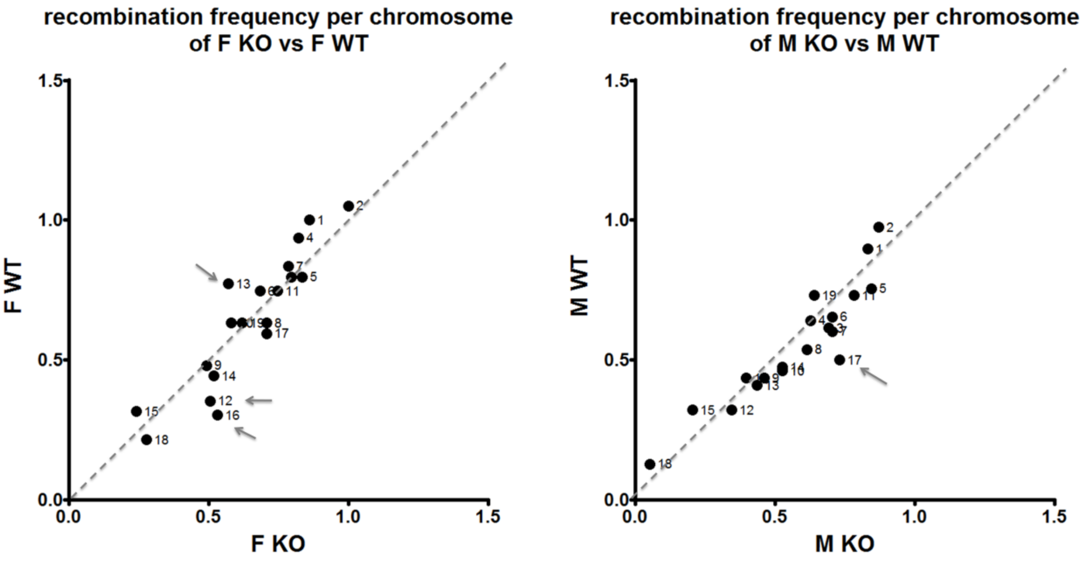

Figure S3 Representation of the average recombination frequency per chromosome of FWT vs FKO and MWT vs MKO study groups. Each dot is a chromosome identified by its number. A bisector line (represented as a grey dashed line) corresponds to equal recombination frequencies between the FWT and FKO or MWT and MKO analysis groups and deviations from this line, even if not significant, were observed for females in Chromosomes 12, 13 and 16 and for males in Chromosome 17 (arrows).

Figure S4.A

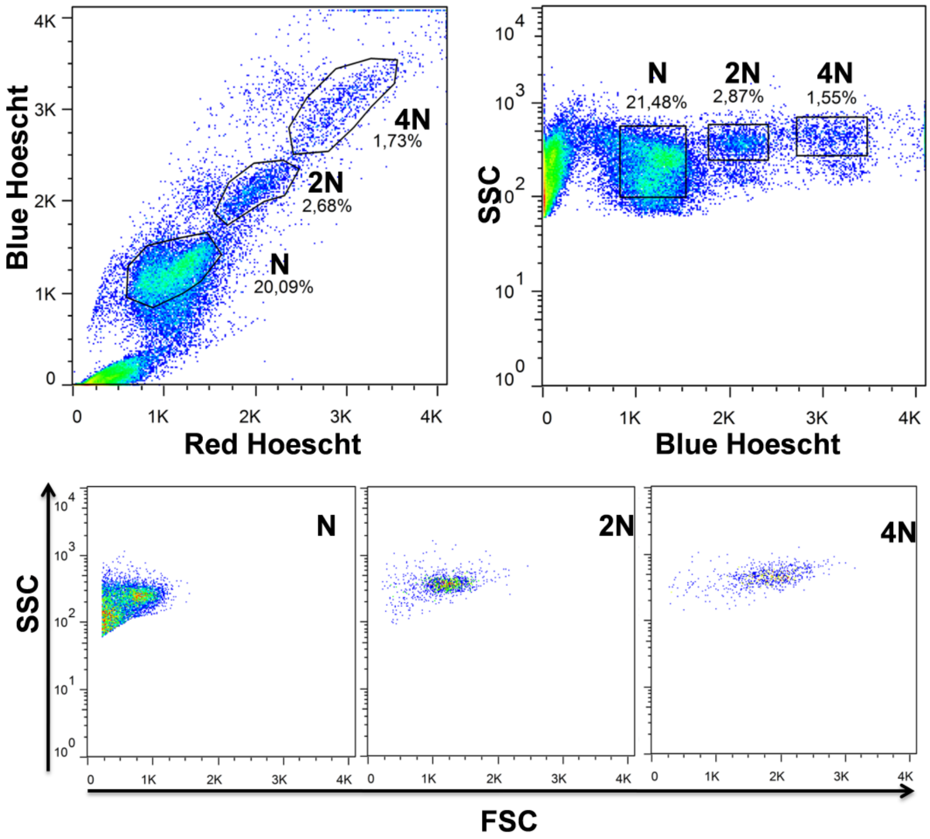

Figure S4.B

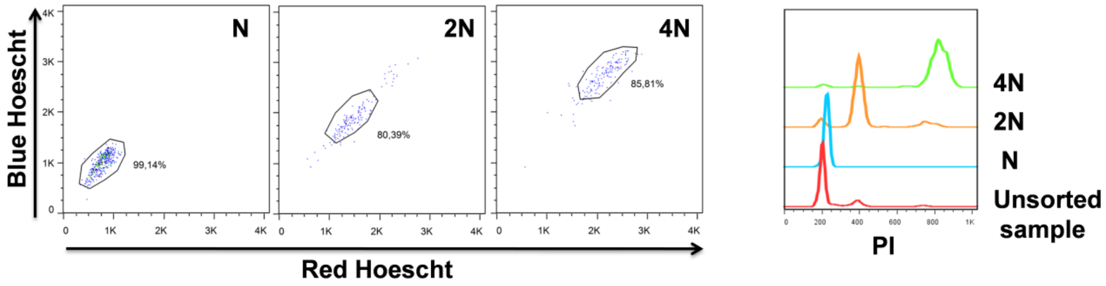

Hoescht	N	2N	4N
Number of values	6	6	5
Minimum	95,89	76,62	75,73
Maximum	99,73	97,73	93,44
Mean	98,47	89,66	87,23
Std. Error	0,60	3,06	3,272

PI incorporation	N	2N	4N
Number of values	6	6	5
Minimum	95,04	71,07	65,82
Maximum	98,89	86,06	95,86
Mean	97,51	78,9	84,78
Std. Error	0,56	2,22	5,932

Figure S4 **(A)** Sorting strategy for subpopulations of testicular sperm cells according to DNA content using Hoechst 33342 - a vital dye that binds to DNA – as previously described (BASTOS et al. 2005). In the 2 top panels, are represented dot-plots with gates on the cell subpopulations to sort and in the bottom panel is represented the FSC-SSC profile of the subpopulations, which is easily identifiable. **(B)** Purity of the sorted populations, measured by acquisition of the subpopulations after sorting (left) and by DNA content measured by PI incorporation (right) and in the table the percentages of purity achieved for all the samples used in the real-time PCR.

Figure S5.

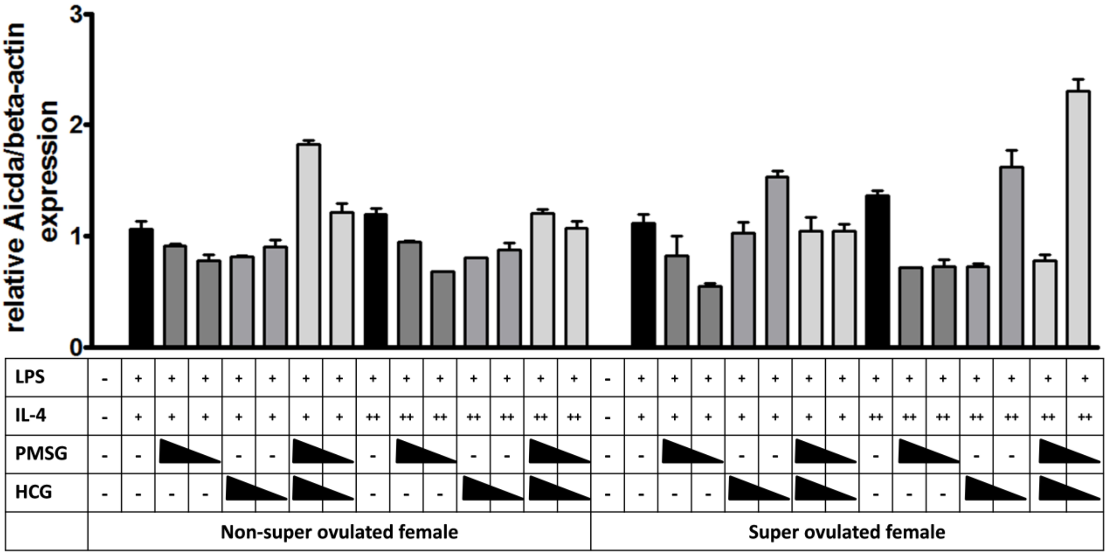

Figure S5 To control for the effect of the hormone stimulation on AID expression, splenic B-cells from super-ovulated and from non-super-ovulated C57BL/6J were stimulated in culture with LPS and under different IL4 concentrations (++ is double concentration of +), with or without PMSG (2.5 and 0.5 IU/ml) and HCG (2.5 and 0.5 IU/ml) or both in the culture medium. AID expression was measured by real-time PCR. Error bars correspond to technical triplicates.

Table S1 Panel of the initial SNPs and of the 130 used SNPs and the calculated distances between the SNP pairs used for the calculation of the recombination frequencies.

SNP ID	CHROMOSOME	LOCATION (bp)	BALB/c	C57Bl/6		DISTANCE BETWEEN TWO SNPs (Mbp)
rs13475717	1	8812450	T	C		
rs6361963	1	22619335	G	A		13.807
rs6369312	1	38667353	C	T		16.048
rs13475863	1	50661113	C	G		11.994
rs3022803	1	67810119	C	A		17.149
rs13475941	1	79657080	T	C		11.847
rs13475991	1	96353648	C	G		16.697
rs13476049	1	112066521	T	C		15.713
rs13476096	1	127739701	A	C		15.673
rs13476148	1	144782263	T	G	a	
rs13476193	1	158100089	A	G		30.360
rs3022859	1	173062911	C	T		14.963
rs13476286	1	187587911	C	T		14.525
rs13476315	1	196278816	A	G		8.691
rs13476334	2	7293211	G	A		
rs13476554	2	67043102	T	A	b	
rs13476605	2	82275273	G	A		74.982
rs13476658	2	98329842	T	C		16.055
rs13476764	2	127839760	C	T		29.510
rs13476869	2	158222762	G	A		30.383
rs13476916	2	170922417	C	G		12.700
rs13476944	2	180856846	A	T		9.934
rs29670066	3	8978594	A	G		
rs13477071	3	40244400	A	G		31.266
rs13477111	3	52924293	A	G		12.680
rs13477284	3	99205144	A	C		46.281
rs13477346	3	113527431	T	C		14.322
rs13477393	3	127821893	C	G		14.294
rs13477461	3	143083217	G	T		15.261
rs13477505	3	154454202	G	A		11.371
rs13477553	4	9390856	C	T		
rs32479301	4	21381965	T	A	b	
rs13477650	4	37673548	G	C		28.283
rs13477707	4	53033101	T	G		15.360
rs13477759	4	68763508	G	A		15.730

rs13477807	4	82473949	G	C		13.710
rs13477857	4	96016884	T	A	a	
rs13477923	4	114333284	G	C		31.859
rs13477977	4	129075835	T	C		14.743
rs13478025	4	140887203	G	A		11.811
rs13478068	4	154061972	G	A		13.175
rs6245801	5	7467346	A	G		
rs13478139	5	22525956	G	A	a	
rs13478250	5	51732341	G	A		44.265
rs4225252	5	66941010	T	C		15.209
rs6319876	5	81554093	A	G		14.613
rs13478409	5	98605005	C	T		17.051
rs13478463	5	112313964	C	T		13.709
rs13478512	5	126563550	G	A		14.250
rs13478567	5	142445658	T	C		15.882
rs13478595	5	150614341	G	A		8.169
rs6206775	6	7458991	T	C		
rs13478661	6	24098235	T	C		16.639
rs3024195	6	36544690	G	A		12.446
rs3719573	6	65358844	T	A		28.814
rs13478859	6	82369651	C	T		17.011
rs13478917	6	97434657	G	T	b	
rs13478975	6	112109489	C	T	b	
rs13479023	6	127404078	G	A	b	
rs13479082	6	143993994	A	G		61.624
rs13479115	7	6807732	C	T		
rs13479149	7	23115448	G	C		16.308
rs13479197	7	37505757	G	T		14.390
rs13479253	7	52740792	A	G		15.235
rs13479313	7	67899711	G	A		15.159
rs13479366	7	81616483	G	A		13.717
rs6175007	7	114685318	C	T		33.069
rs3024212	7	127245370	G	A		12.560
rs13479561	7	139556032	A	T		12.311
rs13479603	8	9530435	C	T		
rs33049139	8	23989783	A	G		14.459
rs13479698	8	36937716	G	A		12.948
rs13479735	8	47062726	C	T		10.125
rs13479802	8	67392271	A	C		20.330
rs13479860	8	85157384	G	A		17.765

rs13479919	8	94844852	T	C		9.687
rs13479986	8	114019724	C	T		19.175
rs13480028	8	127213239	A	G		13.194
rs13480150	9	39474933	T	C		
rs13480196	9	52462952	G	A		12.988
rs13480311	9	83242331	T	G		30.779
rs13480364	9	97989839	C	A		14.748
rs6211405	9	111562171	G	A		13.572
rs13480460	9	122290754	C	G		10.729
rs13480488	10	8934508	A	G		
rs13480540	10	22380249	G	A	a	
rs13480579	10	35887456	C	T		26.953
rs13480617	10	56954219	G	A	b	
rs13480643	10	69957648	G	A	b	
rs13480679	10	84343358	C	T		48.456
rs13480723	10	97377664	C	T		13.034
rs13480770	10	113145621	T	A		15.768
rs6290313	11	8585339	C	T		
rs13480913	11	23633252	T	C		15.048
rs13480971	11	37095883	C	A		13.463
rs13481078	11	67002286	C	T		29.906
rs13481177	11	97596914	G	A		30.595
rs13481239	11	113366849	G	A		15.770
rs6280170	11	120295398	G	A		6.929
rs13481294	12	9596339	C	T	b	
rs4229289	12	25685332	A	G	b	
rs13481394	12	37076846	G	A		
rs13481451	12	52732418	G	A	a/b	
rs13481511	12	68942686	C	T		31.866
rs13481556	12	81581401	T	C		12.639
rs13481636	12	108001267	C	A		26.420
rs29230496	13	9024512	T	A		
rs13459140	13	37995025	C	T		28.971
rs13481815	13	54405468	C	T		16.410
rs3023384	13	68339380	T	C		13.934
rs13481906	13	81922201	T	C		13.583
rs6293514	13	98968792	C	T		17.047
rs13482015	13	112926521	G	A		13.958
rs6364554	14	23783469	C	T		
rs13482159	14	44623181	T	C		20.840

rs13482191	14	53600552	A	G		8.977
rs6284381	14	67085819	G	A		13.485
rs13482284	14	85046715	T	C		17.961
rs13482334	14	98222674	G	A		13.176
rs13482379	14	111758947	G	T		13.536
rs13482407	14	121187820	G	A		9.429
rs13482477	15	22463930	A	G		
rs3666986	15	38738734	G	A		16.275
rs13482577	15	51482848	A	G		12.744
rs13482627	15	67560315	C	T		16.077
rs4230908	15	82954168	C	T		15.394
rs4158907	16	7878150	T	C	b	
rs4165334	16	23382939	C	T		
rs4174174	16	37702077	C	T		14.319
rs4198331	16	68033306	T	C		30.331
rs4211695	16	82915297	C	G		14.882
rs4220176	16	93531942	A	C		10.617
rs13482862	17	8655883	A	G		
rs4231389	17	24962178	G	A	b	
rs3145728	17	37447963	T	C		28.792
rs33319224	17	67934113	T	C		30.486
rs13483144	17	85794298	T	G		17.860
rs13483171	17	92848431	A	G		7.054
rs13483199	18	9736283	G	A		
rs13483247	18	21628769	G	A		11.892
rs13483307	18	38143484	C	T		16.515
rs13483361	18	52245709	C	A		14.102
rs6346101	18	68639844	C	A	b	
rs13483521	19	9020965	G	T		
rs13483566	19	22621254	A	G		13.600
rs13483615	19	37964227	C	A	b	
rs13483670	19	52493446	T	C		29.872
rs6391539	19	60463032	T	C		7.970
Average						18.568
Sum						2061.041

Notes:
a. SNP not used because it did not pass the threshold of 95% of genotype signal (call).
b. SNP not used because it did not pass the analysis of no distortion from the expected 50% inheritance of each allele on a 99% confidence interval.

Table S2 Independent data sets to evaluate first results for recombination between SNP pairs.

		MALE		FEMALE	
CHR.	SNP PAIR APROXIMATE POSITIONS	1ST DATA SET	2ND DATA SET	1ST DATA SET	2ND DATA SET
8	9.5 and 24Mbp	0.167	0.333	0.037	1.000
9	98 and 112Mbp	0.130	0.648	0.765	1.000
13	99 and 113Mbp	1.000	0.801	0.144	0.498
16	23 and 38Mbp	0.719	0.489	0.077	0.592
16	38 and 68Mbp	0.367	0.844	1.000	1.000
16	68 and 86Mbp	0.328	0.281	0.047	0.747
17	68 and 86Mbp	< 0.0001	0.726	1.000	0.541
19	9 and 23Mbp	0.859	0.446	0.024	1.000
19	23 and 53Mbp	1.000	0.331	0.008	1.000

Statistical *p* values of the comparison between analysis groups MWT vs. MKO and FWT vs. FKO for 6 SNP pairs chosen randomly, for 2 SNP pairs (chromosome 19 for FWT vs. FKO analysis) for which in the first analysis the statistical difference was relatively high but not significant and 1SNP pair in chromosome 17 (for MWT vs. MKO analysis) for which the statistical analysis indicated a significant difference (1st data set) and for an independent and larger set of samples from the same matings (2nd data set).

Table S3 Comparison of the percentage of detected recombination events per chromosome in this study compared to an exhaustive analysis.

	THIS STUDY (WT sex-averaged)		REF. STUDY (Shifman *et al*. 2006)	
CHR. #	Average REC	cM/Mbp[a]	cM/Mbp	%
1	0.95	0.51	0.51	99
2	1.01	0.58	0.59	99
3	0.71	0.49	0.52	93
4	0.79	0.55	0.59	92
5	0.78	0.54	0.65	84
6	0.70	0.51	0.60	86
7	0.72	0.54	0.60	90
8	0.59	0.50	0.59	84
9	0.46	0.55	0.66	84
10	0.55	0.53	0.63	83
11	0.74	0.66	0.75	88
12	0.34	0.48	0.62	77
13	0.59	0.57	0.59	96
14	0.46	0.47	0.55	86
15	0.32	0.53	0.68	77
16	0.37	0.53	0.70	75
17	0.55	0.65	0.69	94
18	0.17	0.40	0.81	50
19	0.68	1.33	1.01	131

a. cM/Mpb are calculated using the distances shown in Table 1.
