# Supplementary material for: Activation-Induced Cytidine Deaminase Does Not Impact Murine Meiotic Recombination
Source: G3 (Bethesda). 2013 Apr 1;3(4):645–55. doi: 10.1534/g3.113.005553 (PMC3618351; doi:10.1534/g3.113.005553)
Supplement: Supporting Information [file supp_g3.113.005553_FigureS1.pdf]

Figure S1. A

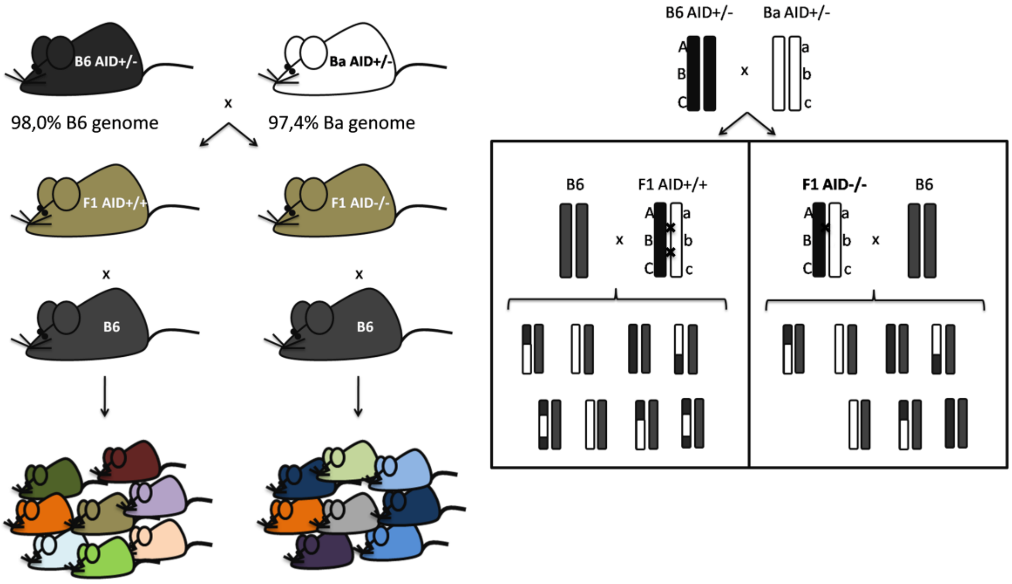

Figure S1. B

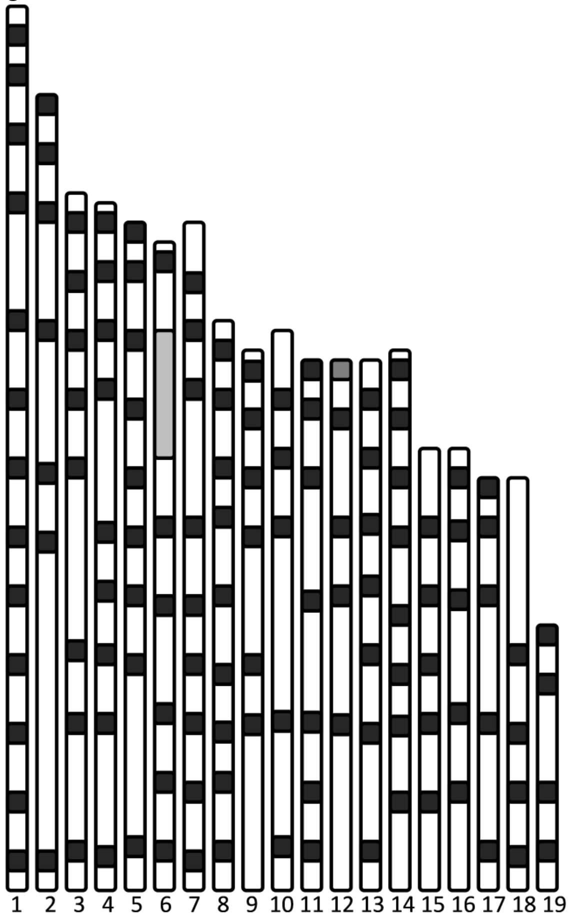

Figure S1 **(A)** Schematics of the mating strategy : matings of B6.Aicda+/- and BA.Aicda+/-; F1 were genotyped for Aicda locus. F1.Aicda-/- and F1.Aicda+/+ were reciprocally mated with C57BL/6J and offspring was genotyped for SNPs that distinguish C57BL/6J from BALB/c genetic background. (B) Schematics of the SNP relative positions on the 19 autossomes in black. In Chromosome 6, the light grey area represents the portion that contains the Aicda locus and that is still of 129 genome. In medium grey is represented the relative position of the Variable Number Tandem Repeat (VNTR) in chromosome 12.
