# Supplementary material for: Activation-Induced Cytidine Deaminase Does Not Impact Murine Meiotic Recombination
Source: G3 (Bethesda). 2013 Apr 1;3(4):645–55. doi: 10.1534/g3.113.005553 (PMC3618351; doi:10.1534/g3.113.005553)
Supplement: Supporting Information [file supp_g3.113.005553_FigureS2.pdf]

Figure S2.

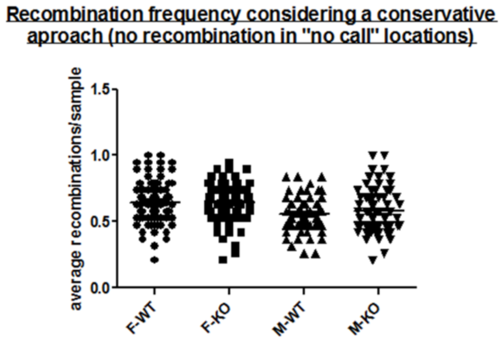

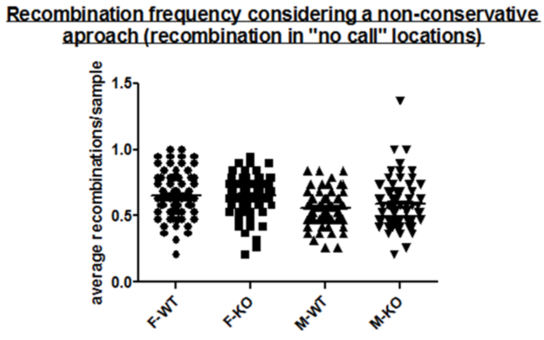

ManWhitey Test	Fwt no rec	Fko no rec	Mwt no rec	Mko no rec
Fwt no rec	na	0,6155	0,0007	0,0046
Fko no rec	na	na	< 0,0001	0,0008
Mwt no rec	na	na	na	0,8192
Mko no rec	na	na	na	na

ManWhitey Test	Fwt rec	Fko rec	Mwt rec	Mko rec
Fwt rec	na	0,6632	0,0004	0,0046
Fko rec	na	na	< 0,0001	0,0008
Mwt rec	na	na	na	0,7264
Mko rec	na	na	na	na

Figure S2 For the 17 SNP/sample pairs that passed the selection filters but had no detectable genotype signal, two extreme scenarios (recombination in all locations versus no recombination in all locations) were considered and overall recombination frequencies were calculated for the two extreme scenarios.
