# Supplementary material for: Activation-Induced Cytidine Deaminase Does Not Impact Murine Meiotic Recombination
Source: G3 (Bethesda). 2013 Apr 1;3(4):645–55. doi: 10.1534/g3.113.005553 (PMC3618351; doi:10.1534/g3.113.005553)
Supplement: Supporting Information [file supp_g3.113.005553_FigureS3.pdf]

Figure S3.

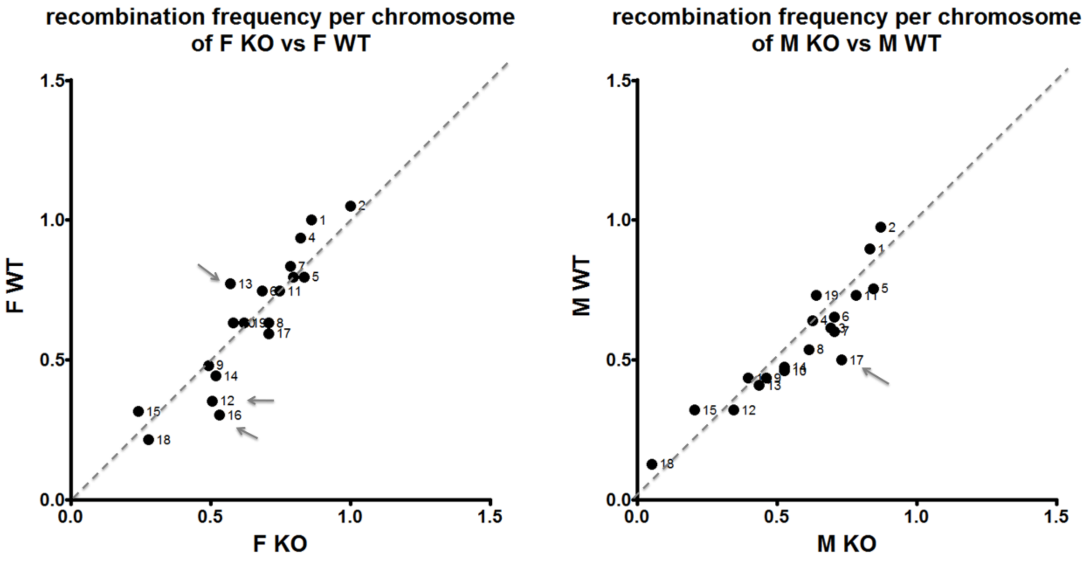

Figure S3 Representation of the average recombination frequency per chromosome of FWT vs FKO and MWT vs MKO study groups. Each dot is a chromosome identified by its number. A bisector line (represented as a grey dashed line) corresponds to equal recombination frequencies between the FWT and FKO or MWT and MKO analysis groups and deviations from this line, even if not significant, were observed for females in Chromosomes 12, 13 and 16 and for males in Chromosome 17 (arrows).
