# Supplementary material for: Activation-Induced Cytidine Deaminase Does Not Impact Murine Meiotic Recombination
Source: G3 (Bethesda). 2013 Apr 1;3(4):645–55. doi: 10.1534/g3.113.005553 (PMC3618351; doi:10.1534/g3.113.005553)
Supplement: Supporting Information [file supp_g3.113.005553_FigureS4.pdf]

Figure S4.A

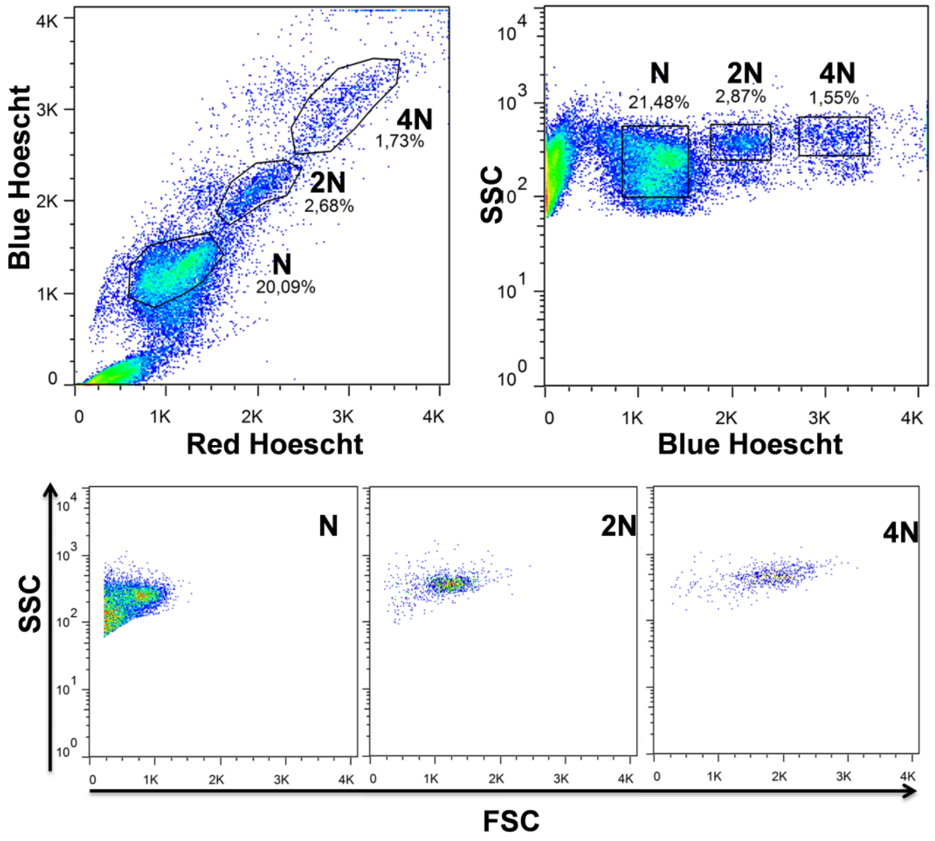

Figure S4.B

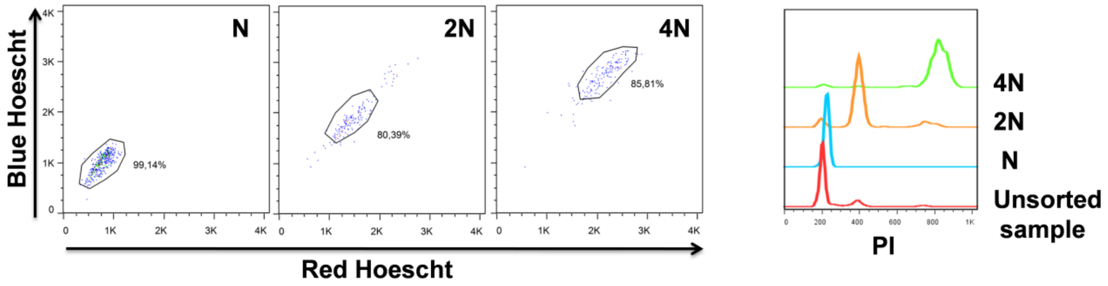

Hoescht	N	2N	4N
Number of values	6	6	5
Minimum	95,89	76,62	75,73
Maximum	99,73	97,73	93,44
Mean	98,47	89,66	87,23
Std. Error	0,60	3,06	3,272

PI incorporation	N	2N	4N
Number of values	6	6	5
Minimum	95,04	71,07	65,82
Maximum	98,89	86,06	95,86
Mean	97,51	78,9	84,78
Std. Error	0,56	2,22	5,932

Figure S4 **(A)** Sorting strategy for subpopulations of testicular sperm cells according to DNA content using Hoechst 33342 - a vital dye that binds to DNA – as previously described (BASTOS et al. 2005). In the 2 top panels, are represented dot-plots with gates on the cell subpopulations to sort and in the bottom panel is represented the FSC-SSC profile of the subpopulations, which is easily identifiable. **(B)** Purity of the sorted populations, measured by acquisition of the subpopulations after sorting (left) and by DNA content measured by PI incorporation (right) and in the table the percentages of purity achieved for all the samples used in the real-time PCR.
