# Supplementary material for: Activation-Induced Cytidine Deaminase Does Not Impact Murine Meiotic Recombination
Source: G3 (Bethesda). 2013 Apr 1;3(4):645–55. doi: 10.1534/g3.113.005553 (PMC3618351; doi:10.1534/g3.113.005553)
Supplement: Supporting Information [file supp_g3.113.005553_FigureS5.pdf]

Figure S5.

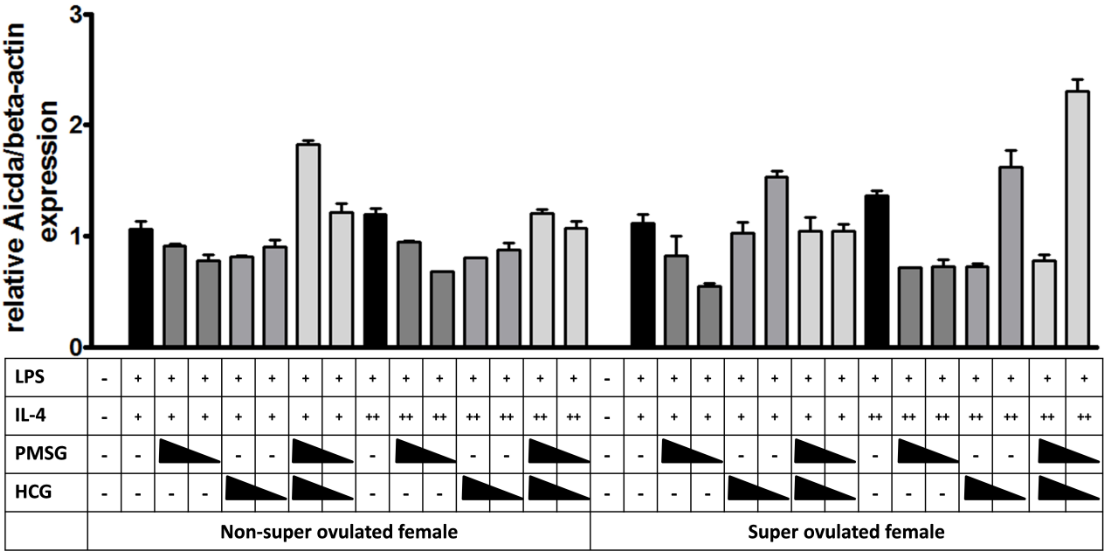

Figure S5 To control for the effect of the hormone stimulation on AID expression, splenic B-cells from super-ovulated and from non-super-ovulated C57BL/6J were stimulated in culture with LPS and under different IL4 concentrations (++ is double concentration of +), with or without PMSG (2.5 and 0.5 IU/ml) and HCG (2.5 and 0.5 IU/ml) or both in the culture medium. AID expression was measured by real-time PCR. Error bars correspond to technical triplicates.
