# Supplementary material for: Activation-Induced Cytidine Deaminase Does Not Impact Murine Meiotic Recombination
Source: G3 (Bethesda). 2013 Apr 1;3(4):645–55. doi: 10.1534/g3.113.005553 (PMC3618351; doi:10.1534/g3.113.005553)
Supplement: Supporting Information [file supp_g3.113.005553_TableS1.pdf]

Table S1 Panel of the initial SNPs and of the 130 used SNPs and the calculated distances between the SNP pairs used for the calculation of the recombination frequencies.

SNP ID	CHROMOSOME	LOCATION (bp)	BALB/c	C57Bl/6		DISTANCE BETWEEN TWO SNPs (Mbp)
rs13475717	1	8812450	T	C		
rs6361963	1	22619335	G	A		13.807
rs6369312	1	38667353	C	T		16.048
rs13475863	1	50661113	C	G		11.994
rs3022803	1	67810119	C	A		17.149
rs13475941	1	79657080	T	C		11.847
rs13475991	1	96353648	C	G		16.697
rs13476049	1	112066521	T	C		15.713
rs13476096	1	127739701	A	C		15.673
rs13476148	1	144782263	T	G	a	
rs13476193	1	158100089	A	G		30.360
rs3022859	1	173062911	C	T		14.963
rs13476286	1	187587911	C	T		14.525
rs13476315	1	196278816	A	G		8.691
rs13476334	2	7293211	G	A		
rs13476554	2	67043102	T	A	b	
rs13476605	2	82275273	G	A		74.982
rs13476658	2	98329842	T	C		16.055
rs13476764	2	127839760	C	T		29.510
rs13476869	2	158222762	G	A		30.383
rs13476916	2	170922417	C	G		12.700
rs13476944	2	180856846	A	T		9.934
rs29670066	3	8978594	A	G		
rs13477071	3	40244400	A	G		31.266
rs13477111	3	52924293	A	G		12.680
rs13477284	3	99205144	A	C		46.281
rs13477346	3	113527431	T	C		14.322
rs13477393	3	127821893	C	G		14.294
rs13477461	3	143083217	G	T		15.261
rs13477505	3	154454202	G	A		11.371
rs13477553	4	9390856	C	T		
rs32479301	4	21381965	T	A	b	
rs13477650	4	37673548	G	C		28.283
rs13477707	4	53033101	T	G		15.360
rs13477759	4	68763508	G	A		15.730

rs13477807	4	82473949	G	C		13.710
rs13477857	4	96016884	T	A	a	
rs13477923	4	114333284	G	C		31.859
rs13477977	4	129075835	T	C		14.743
rs13478025	4	140887203	G	A		11.811
rs13478068	4	154061972	G	A		13.175
rs6245801	5	7467346	A	G		
rs13478139	5	22525956	G	A	a	
rs13478250	5	51732341	G	A		44.265
rs4225252	5	66941010	T	C		15.209
rs6319876	5	81554093	A	G		14.613
rs13478409	5	98605005	C	T		17.051
rs13478463	5	112313964	C	T		13.709
rs13478512	5	126563550	G	A		14.250
rs13478567	5	142445658	T	C		15.882
rs13478595	5	150614341	G	A		8.169
rs6206775	6	7458991	T	C		
rs13478661	6	24098235	T	C		16.639
rs3024195	6	36544690	G	A		12.446
rs3719573	6	65358844	T	A		28.814
rs13478859	6	82369651	C	T		17.011
rs13478917	6	97434657	G	T	b	
rs13478975	6	112109489	C	T	b	
rs13479023	6	127404078	G	A	b	
rs13479082	6	143993994	A	G		61.624
rs13479115	7	6807732	C	T		
rs13479149	7	23115448	G	C		16.308
rs13479197	7	37505757	G	T		14.390
rs13479253	7	52740792	A	G		15.235
rs13479313	7	67899711	G	A		15.159
rs13479366	7	81616483	G	A		13.717
rs6175007	7	114685318	C	T		33.069
rs3024212	7	127245370	G	A		12.560
rs13479561	7	139556032	A	T		12.311
rs13479603	8	9530435	C	T		
rs33049139	8	23989783	A	G		14.459
rs13479698	8	36937716	G	A		12.948
rs13479735	8	47062726	C	T		10.125
rs13479802	8	67392271	A	C		20.330
rs13479860	8	85157384	G	A		17.765

rs13479919	8	94844852	T	C		9.687
rs13479986	8	114019724	C	T		19.175
rs13480028	8	127213239	A	G		13.194
rs13480150	9	39474933	T	C		
rs13480196	9	52462952	G	A		12.988
rs13480311	9	83242331	T	G		30.779
rs13480364	9	97989839	C	A		14.748
rs6211405	9	111562171	G	A		13.572
rs13480460	9	122290754	C	G		10.729
rs13480488	10	8934508	A	G		
rs13480540	10	22380249	G	A	a	
rs13480579	10	35887456	C	T		26.953
rs13480617	10	56954219	G	A	b	
rs13480643	10	69957648	G	A	b	
rs13480679	10	84343358	C	T		48.456
rs13480723	10	97377664	C	T		13.034
rs13480770	10	113145621	T	A		15.768
rs6290313	11	8585339	C	T		
rs13480913	11	23633252	T	C		15.048
rs13480971	11	37095883	C	A		13.463
rs13481078	11	67002286	C	T		29.906
rs13481177	11	97596914	G	A		30.595
rs13481239	11	113366849	G	A		15.770
rs6280170	11	120295398	G	A		6.929
rs13481294	12	9596339	C	T	b	
rs4229289	12	25685332	A	G	b	
rs13481394	12	37076846	G	A		
rs13481451	12	52732418	G	A	a/b	
rs13481511	12	68942686	C	T		31.866
rs13481556	12	81581401	T	C		12.639
rs13481636	12	108001267	C	A		26.420
rs29230496	13	9024512	T	A		
rs13459140	13	37995025	C	T		28.971
rs13481815	13	54405468	C	T		16.410
rs3023384	13	68339380	T	C		13.934
rs13481906	13	81922201	T	C		13.583
rs6293514	13	98968792	C	T		17.047
rs13482015	13	112926521	G	A		13.958
rs6364554	14	23783469	C	T		
rs13482159	14	44623181	T	C		20.840

rs13482191	14	53600552	A	G		8.977
rs6284381	14	67085819	G	A		13.485
rs13482284	14	85046715	T	C		17.961
rs13482334	14	98222674	G	A		13.176
rs13482379	14	111758947	G	T		13.536
rs13482407	14	121187820	G	A		9.429
rs13482477	15	22463930	A	G		
rs3666986	15	38738734	G	A		16.275
rs13482577	15	51482848	A	G		12.744
rs13482627	15	67560315	C	T		16.077
rs4230908	15	82954168	C	T		15.394
rs4158907	16	7878150	T	C	b	
rs4165334	16	23382939	C	T		
rs4174174	16	37702077	C	T		14.319
rs4198331	16	68033306	T	C		30.331
rs4211695	16	82915297	C	G		14.882
rs4220176	16	93531942	A	C		10.617
rs13482862	17	8655883	A	G		
rs4231389	17	24962178	G	A	b	
rs3145728	17	37447963	T	C		28.792
rs33319224	17	67934113	T	C		30.486
rs13483144	17	85794298	T	G		17.860
rs13483171	17	92848431	A	G		7.054
rs13483199	18	9736283	G	A		
rs13483247	18	21628769	G	A		11.892
rs13483307	18	38143484	C	T		16.515
rs13483361	18	52245709	C	A		14.102
rs6346101	18	68639844	C	A	b	
rs13483521	19	9020965	G	T		
rs13483566	19	22621254	A	G		13.600
rs13483615	19	37964227	C	A	b	
rs13483670	19	52493446	T	C		29.872
rs6391539	19	60463032	T	C		7.970
Average						18.568
Sum						2061.041

Notes:
a. SNP not used because it did not pass the threshold of 95% of genotype signal (call).
b. SNP not used because it did not pass the analysis of no distortion from the expected 50% inheritance of each allele on a 99% confidence interval.
