# Supplementary material for: Activation-Induced Cytidine Deaminase Does Not Impact Murine Meiotic Recombination
Source: G3 (Bethesda). 2013 Apr 1;3(4):645–55. doi: 10.1534/g3.113.005553 (PMC3618351; doi:10.1534/g3.113.005553)
Supplement: Supporting Information [file supp_g3.113.005553_TableS2.pdf]

Table S2 Independent data sets to evaluate first results for recombination between SNP pairs.

		MALE		FEMALE	
CHR.	SNP PAIR APROXIMATE POSITIONS	1ST DATA SET	2ND DATA SET	1ST DATA SET	2ND DATA SET
8	9.5 and 24Mbp	0.167	0.333	0.037	1.000
9	98 and 112Mbp	0.130	0.648	0.765	1.000
13	99 and 113Mbp	1.000	0.801	0.144	0.498
16	23 and 38Mbp	0.719	0.489	0.077	0.592
16	38 and 68Mbp	0.367	0.844	1.000	1.000
16	68 and 86Mbp	0.328	0.281	0.047	0.747
17	68 and 86Mbp	< 0.0001	0.726	1.000	0.541
19	9 and 23Mbp	0.859	0.446	0.024	1.000
19	23 and 53Mbp	1.000	0.331	0.008	1.000

Statistical *p* values of the comparison between analysis groups MWT vs. MKO and FWT vs. FKO for 6 SNP pairs chosen randomly, for 2 SNP pairs (chromosome 19 for FWT vs. FKO analysis) for which in the first analysis the statistical difference was relatively high but not significant and 1SNP pair in chromosome 17 (for MWT vs. MKO analysis) for which the statistical analysis indicated a significant difference (1st data set) and for an independent and larger set of samples from the same matings (2nd data set).
