# Supplementary material for: Activation-Induced Cytidine Deaminase Does Not Impact Murine Meiotic Recombination
Source: G3 (Bethesda). 2013 Apr 1;3(4):645–55. doi: 10.1534/g3.113.005553 (PMC3618351; doi:10.1534/g3.113.005553)
Supplement: Supporting Information [file supp_g3.113.005553_TableS3.pdf]

Table S3   Comparison of the percentage of detected recombination events per chromosome in this study compared to an exhaustive analysis.

	THIS STUDY (WT sex-averaged)		REF. STUDY (Shifman *et al*. 2006)	
CHR. #	Average REC	cM/Mbp[a]	cM/Mbp	%
1	0.95	0.51	0.51	99
2	1.01	0.58	0.59	99
3	0.71	0.49	0.52	93
4	0.79	0.55	0.59	92
5	0.78	0.54	0.65	84
6	0.70	0.51	0.60	86
7	0.72	0.54	0.60	90
8	0.59	0.50	0.59	84
9	0.46	0.55	0.66	84
10	0.55	0.53	0.63	83
11	0.74	0.66	0.75	88
12	0.34	0.48	0.62	77
13	0.59	0.57	0.59	96
14	0.46	0.47	0.55	86
15	0.32	0.53	0.68	77
16	0.37	0.53	0.70	75
17	0.55	0.65	0.69	94
18	0.17	0.40	0.81	50
19	0.68	1.33	1.01	131

a. cM/Mpb are calculated using the distances shown in Table 1.
